# Cytoplasmic calcium increase via fusion with inactivated Sendai virus induces apoptosis in human multiple myeloma cells by downregulation of c-Myc oncogene

**DOI:** 10.18632/oncotarget.9105

**Published:** 2016-04-29

**Authors:** Yingzhe Jiang, Kotaro Saga, Yasuhide Miyamoto, Yasufumi Kaneda

**Affiliations:** ^1^ Division of Gene Therapy Science, Graduate School of Medicine, Osaka University, Osaka, Japan; ^2^ Department of Immunology, Osaka Medical Center for Cancer and Cardiovascular Diseases, Osaka, Japan

**Keywords:** HVJ-E, apoptosis, Ca^2+^, c-Myc, multiple myeloma

## Abstract

Because the emergence of drug resistance is a major limitation of current treatments for multiple myeloma (MM), it is necessary to continuously develop novel anticancer strategies. Here, using an inactivated Sendai virus (Hemagglutinating Virus of Japan; HVJ) envelope (HVJ-E), we discovered that increase of cytoplasmic Ca^2+^ by virus-cell fusion significantly induced apoptosis against human MM cells but not peripheral blood mononuclear cells from healthy donors. Interaction of F protein of HVJ-E with MM cells increased intracellular Ca^2+^ level of MMs by the induction of Ca^2+^ efflux from endoplasmic reticulum but not influx from extracellular region. The elevation of the Ca^2+^ cytoplasmic level induced SMAD1/5/8 phosphorylation and translocation into the nucleus, and SMAD1/5/8 and SMAD4 complex suppressed c-Myc transcription. Meanwhile, HVJ-E decreases S62 phosphorylation of c-Myc and promotes c-Myc protein degradation. Thus, HVJ-E-induced cell death of MM resulted from suppression of c-Myc by both destabilization of c-Myc protein and downregulation of c-Myc transcription. This study indicates that HVJ-E will be a promising tool for MM therapy.

## INTRODUCTION

Multiple myeloma (MM) is a kind of hematologic B cell malignancy that is featured by the accumulation of abnormal plasma cells in the bone marrow. Current treatments, including dexamethasone, chemotherapy, stem cell transplantation, proteasome inhibitors and immunomodulatory drugs, have made substantial progress toward increasing longevity in MM patients [1, 2]. However, one major limitation of the existing drugs is the eventual emergence of drug resistance for nearly all patients, regardless of the treatment regimen or the initial response to treatment [3]. It is well known that bone marrow microenvironment changes, such as cell adhesion and cytokine-related induction of the JAK/STAT and PI3K/AKT pathways, are responsible for resistance to conventional and novel therapies [4, 5]. Because MM is characterized by a high level of genomic instability and complex cytogenetic aberrations [6, 7], many of these alterations, which include chromosomal translocation, methylation, gene mutation, and microRNA (miRNA) abnormalities, can contribute to drug resistance [3, 8, 9]. Moreover the aberrant expression of oncogenes, such as c-MAF, c-Myc, and cyclin D has been found to contribute to drug resistance in MM [10–12]. Therefore, there is a need to develop new therapeutic strategies for the treatment of MM.

Myc pathway activation increases with the disease stage in MM, and a conditional *MYC* transgene murine model develops a plasma cell malignancy during the late stages of B cell differentiation that shares clinically relevant features of MM [13]. This result supports the central role of c-Myc activity in the pathogenesis of MM. In addition, c-Myc overexpression occurs via different mechanisms, including gene amplification, translocation and mutation. c-Myc chromosomal rearrangements were found in 15% of newly diagnosed MM patients [14], nearly 50% of advanced MM patients [15, 16], 55% of human MM cell lines [14], and such rearrangements have also been implicated in drug resistance in MM [11]. Furthermore, the inhibition of c-Myc activity by a short-hairpin RNA targeting c-Myc has been shown to be lethal to a number of human myeloma cell lines [17]. In addition, a small molecule inhibitor of BRD4 that suppresses c-Myc transcription shows therapeutic effects against MM *in vitro* and *in vivo*, and the small molecule inhibitor 10058-F4a, that specifically inhibits c-Myc-Max heterodimerization, prevents the transactivation of c-Myc, induces cell death in human myeloma cell lines and primary cells of myeloma patients [18, 19]. Thus, c-Myc is likely a promising therapeutic target for MM treatment.

Virotherapy has been shown to have anticancer effects on cancer cells but not normal cells in solid and hematological malignancies. MM represents an attractive target for virotherapy in comparison to many solid tumors because viruses can easily access the tumor cells in the bone marrow and bloodstream. Currently, four RNA viruses (measles virus, vesicular stomatitis virus, reovirus and coxsackievirus A21) and two DNA viruses (adenovirus and vaccinia virus) have shown oncolytic potential against MM [20]. These viruses kill cancer cells via selective viral replication in cancer cells but lose their oncolytic activity following inactivation [21, 22]. To date, inactive viral particles have not been included in oncolytic virotherapy for multiple myeloma.

The Sendai virus (Hemagglutinating Virus of Japan; HVJ) belongs to the paramyxovirus family and has six viral proteins encoded by a negative-sense single-strand RNA genome: nucleocapsid (N); phosphoprotein (P); large (L) matrix (M); fusion (F); and hemagglutinin-neuraminidase (HN). When the Sendai viral genome was cleaved into small pieces by UV irradiation, the viral proteins remained functional [23]. We have documented that the inactivated Sendai virus envelope (HVJ-E) has multiple anti-tumor effects, including the activation of anti-tumor immunity and induction of cancer cell-selective apoptosis [24–26]. Moreover, we demonstrated that HVJ-E RNA fragment-dependent activation of the cytoplasmic RNA receptor retinoic acid-inducible gene-I (RIG-I) and mitochondrial antiviral signaling protein (MAVS) activates the upregulation of TRAIL and Noxa, which enables HVJ-E-mediated cancer-selective apoptosis [26]. The therapeutic potential of HVJ-E has been demonstrated in solid tumors [27–30], but there are no reports concerning whether this virus can be used for the treatment of hematopoietic malignances.

In the present study, we showed that HVJ-E induced cancer-selective apoptosis in multiple myeloma cells with c-Myc addiction. In addition to decrease the phosphorylation of c-Myc at S62 and promote c-Myc protein degradation, HVJ-E induced fusion-mediated elevation of cytoplasmic Ca^2+^ results in c-Myc transcriptional inhibition. Furthermore, we addressed the mechanisms behind cytoplasmic Ca^2+^ increasing induced suppression of c-Myc transcription, activation of SMADs family involved in c-Myc downregulation. These results also provide the possibility that HVJ-E will be a therapeutic tool for MMs.

## RESULTS

### HVJ-E induces apoptosis in human multiple myeloma cell lines but not in PBMCs from healthy donors

To investigate whether HVJ-E exhibits an anti-cancer effect against MM, human myeloma cell lines (NCI-H929, MM1S, RPMI-8226 and U266) and normal human peripheral blood mononuclear cells (PBMCs) from healthy donors were treated with HVJ-E for 72 hours, and cell viability was measured using the MTS assay (Supplementary Figure 1A–1E). The cell viability of NCI-H929, MM1S, and RPMI-8226, but not U266 and PBMCs, was significantly decreased with HVJ-E in a dose-dependent manner. We showed that HVJ-E exhibited cytotoxicity against various MM cells but not healthy PBMCs. Human umbilical vein endothelial cells (HUVECs) and human aortic endothelial cells (HAECs) were also treated with HVJ-E, and the proliferation of HUVECs and HAECs was not inhibited (Supplementary Figure 1F–1G), which is consistent with our previous report [31].

**Figure 1:**
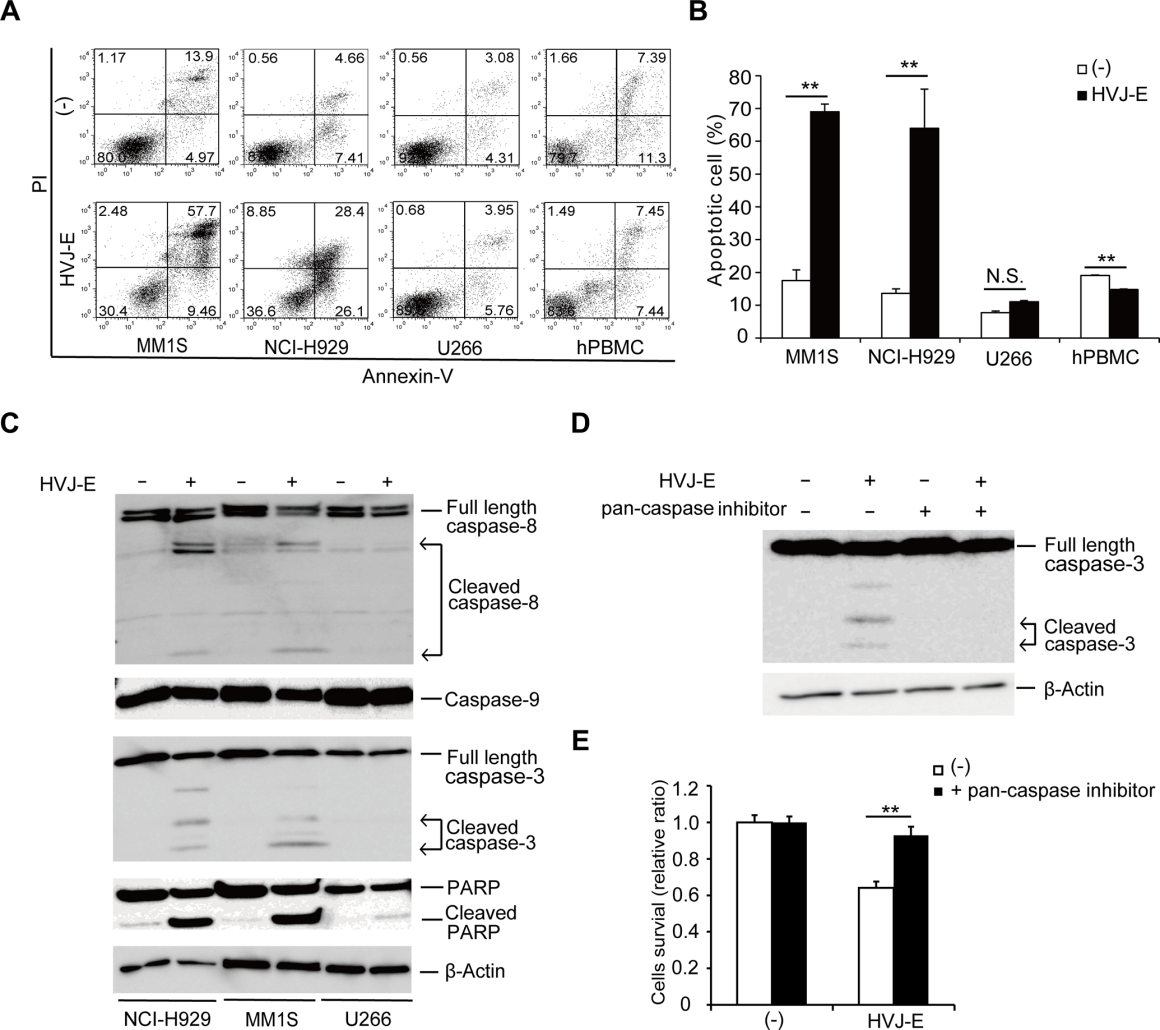
HVJ-E induces caspase-dependent apoptosis in MM cells (**A**) Apoptotic cells were measured in HVJ-E-treated NCI-H929, MM1S, U266 and PBMCs from healthy donors at a multiplicity of infection (MOI) of 1000 for 48 hours using PI (vertical) and Annexin V (horizontal) double staining analysis by FACS. (**B**) Quantification plot of the ratio of the apoptotic (Annexin V^+^) cells shown in Figure 1A. The data represent the mean ± SD of three independent experiments (**P < 0.01, Student's unpaired *t*-test). (**C**) Detection of caspases (caspase-8, -3, -9) and poly (ADP-ribose) polymerase (PARP) in MM cells (NCI-H929, U266, MM1S) after treatment with HVJ-E at an MOI of 1000 for 48 hours by western blotting analysis. (**D**) Immunoblotting results for caspase-3 cleavage after HVJ-E treatment (1000 MOI, 24 hours) in the presence or absence of pan-caspase inhibitor in NCI-H929 cells. (**E**) NCI-H929 cells were treated with HVJ-E at an MOI of 1000 for 48 hours in the presence or absence of pan-caspase inhibitor, and cell viability was assessed using the MTS assay. The data represent the mean ± SD of three independent experiments (**P < 0.01, Student's unpaired *t*-test).

Next, to investigate whether apoptosis is the mechanism underlying HVJ-E-mediated cell death in MM, we assayed certain markers of apoptosis in HVJ-E-treated MMs and PBMCs. Large populations of HVJ-E-treated NCI-H929 and MM1S cells but not U266 cells and PBMCs were Annexin-V-positive (Figure 1A and 1B). Further analysis of leukocyte subsets in PBMCs from healthy donors showed that the Annexin-V-positive apoptotic cells did not change in the CD3^+^ T cell, CD19^+^ B cell and CD14^+^ monocyte populations after HVJ-E treatment (Supplementary Figure 2A and 2B). Although CD15^+^ neutrophils undergo spontaneous apoptosis after 48 hours of incubation *in vitro*, as reported in previous studies [32], no significant difference in CD15^+^Annexin-V^+^ cells was observed in the presence and absence of 48 hours of HVJ-E treatment in neutrophils (Supplementary Figure 2C and 2D). We also examined apoptotic cells among HUVECs and HAECs in the presence and absence of wild-type (WT) and HN-depleted HVJ-E. As shown in Supplementary Figure 2E and 2F, the ratio of Annexin-V-labeled apoptotic cells was not changed by treatment with either WT-HVJ-E or HN-depleted HVJ-E. These results suggest that HVJ-E elicits no harmful effect on healthy leukocytes and endothelial cells after systemic administration. Moreover, the cleavage of caspase-8, caspase-3 and poly(ADP-ribose) polymerase (PARP) was detected in HVJ-E-treated NCI-H929 and MM1S cells but not U266 cells by western blotting analysis (Figure 1C). A pan-caspase inhibitor (Z-VAD-FMK) prevented HVJ-E-mediated activation of caspase-3 (Figure 1D) and cell death (Figure 1E), indicating that HVJ-E induced caspase-dependent apoptosis in MM.

Furthermore, we investigated various proteins related to apoptosis and the expression levels of antiapoptotic Bcl-2 family members B-cell lymphoma-extra-large (Bcl-xL) and B-cell lymphoma 2 (Bcl-2) remained unchanged in responses to HVJ-E treatment in NCI-H929 and MM1S cells (Supplementary Figure 3A). However, we demonstrated that HVJ-E treatment enhanced the expression of the proapoptotic protein Bcl-2-like protein 11 (Bim), which consists of three major isoforms as a result of alternative splicing (BimEL, BimL and BimS), but not BH3 interacting-domain death agonist (Bid), Bcl-2-associated death promoter (Bad), Bcl-2-associated X protein (Bax) or p53-upregulated modulator of apoptosis (Puma) in NCI-H929 cells in a time-dependent manner (Supplementary Figure 3B). By immunoblotting analysis, we also found that HVJ-E induced Bim upregulation in RPMI-8226 and MM1S cell lines (Supplementary Figure 3C). Moreover, HVJ-E-mediated cell death was attenuated by Bim knockdown (Supplementary Figure 3D), suggesting that the Bim upregulation stimulated HVJ-E-induced apoptosis in myeloma cells.

### *In vivo* MM tumor suppression by HVJ-E treatment

**Figure 2:**
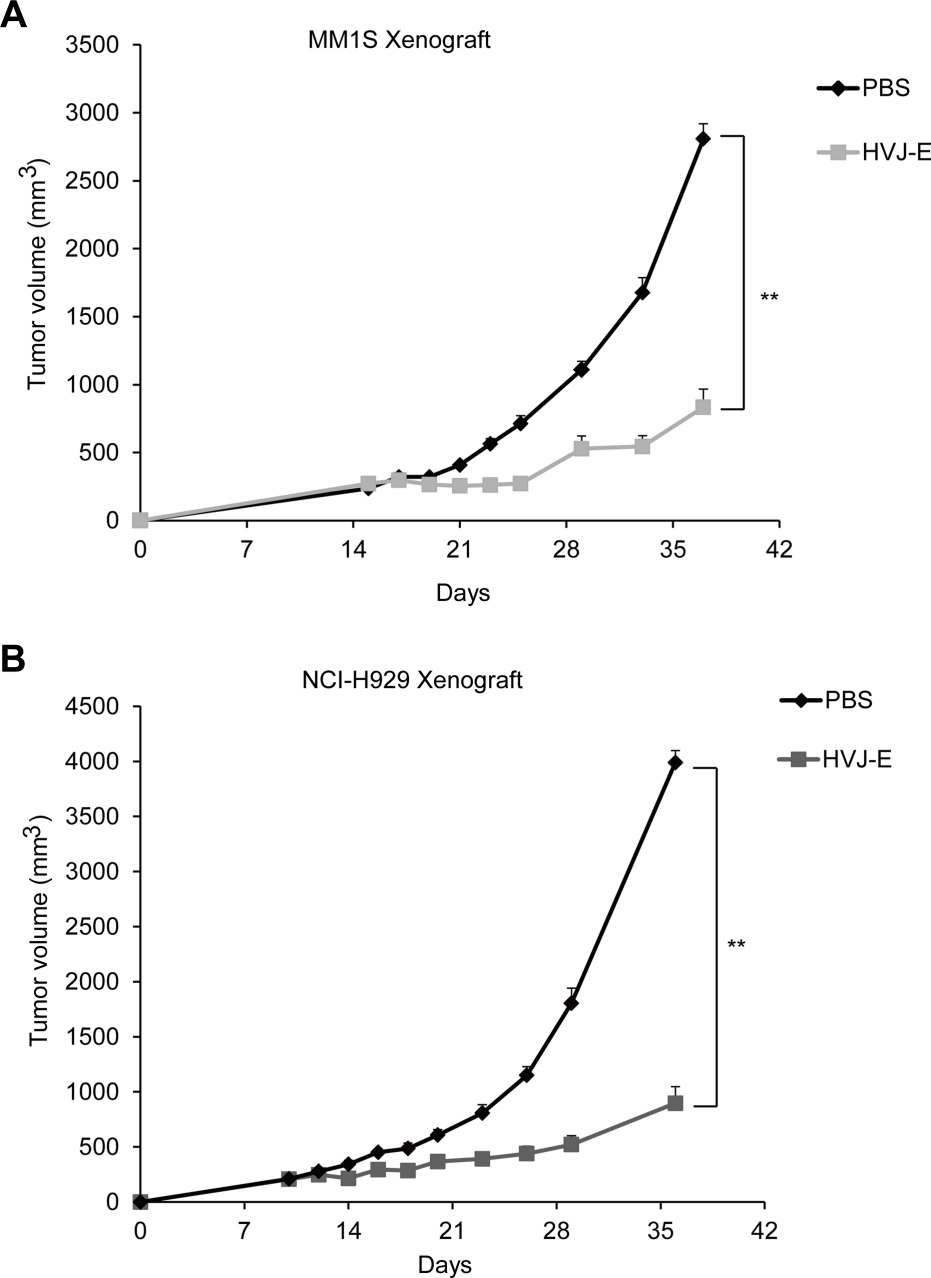
HVJ-E inhibits MM cell growth *in vivo* in MM xenograft mouse models Tumor growth curves were measured in MM1S- (**A**) and NCI-H929-bearing (**B**) NOG mice. The tumor volume (mm^3^) (average and SEM) for each group of mice (*n* = 4–5/group) is shown. The mice were treated with PBS or 5,000 HAU of HVJ-E 3 times per week. A significant delay in tumor growth was noted in HVJ-E-treated mice in comparison to PBS-treated control mice (**P < 0.01, Student's unpaired *t*-test). The bars represent the mean ± SEM. Similar experiments were repeated twice, and the same results were obtained.

To investigate the efficacy of HVJ-E against MM *in vivo*, we incubated an MM1S xenograft in NOG mice. As shown in Figure 2A, HVJ-E treatment substantially lowered the tumor burden in comparison to the PBS group. Moreover, we designed an NCI-H929 xenograft model in NOG mice. NCI-H929 is a human MM cell line derived from a right pleural effusion of a 62-year-old patient who was treated for relapse after treatment with vincristine, melphalan, cytoxan and prednisone [33]. As shown in Figure 2B, HVJ-E treatment significantly suppressed tumor growth in the NCI-H929 xenograft in comparison to PBS treatment. Taken together, these results further support the potential of HVJ-E for the treatment of MM.

### Increases in cytosolic free calcium contribute to HVJ-E-mediated cytotoxicity in MM cells

**Figure 3:**
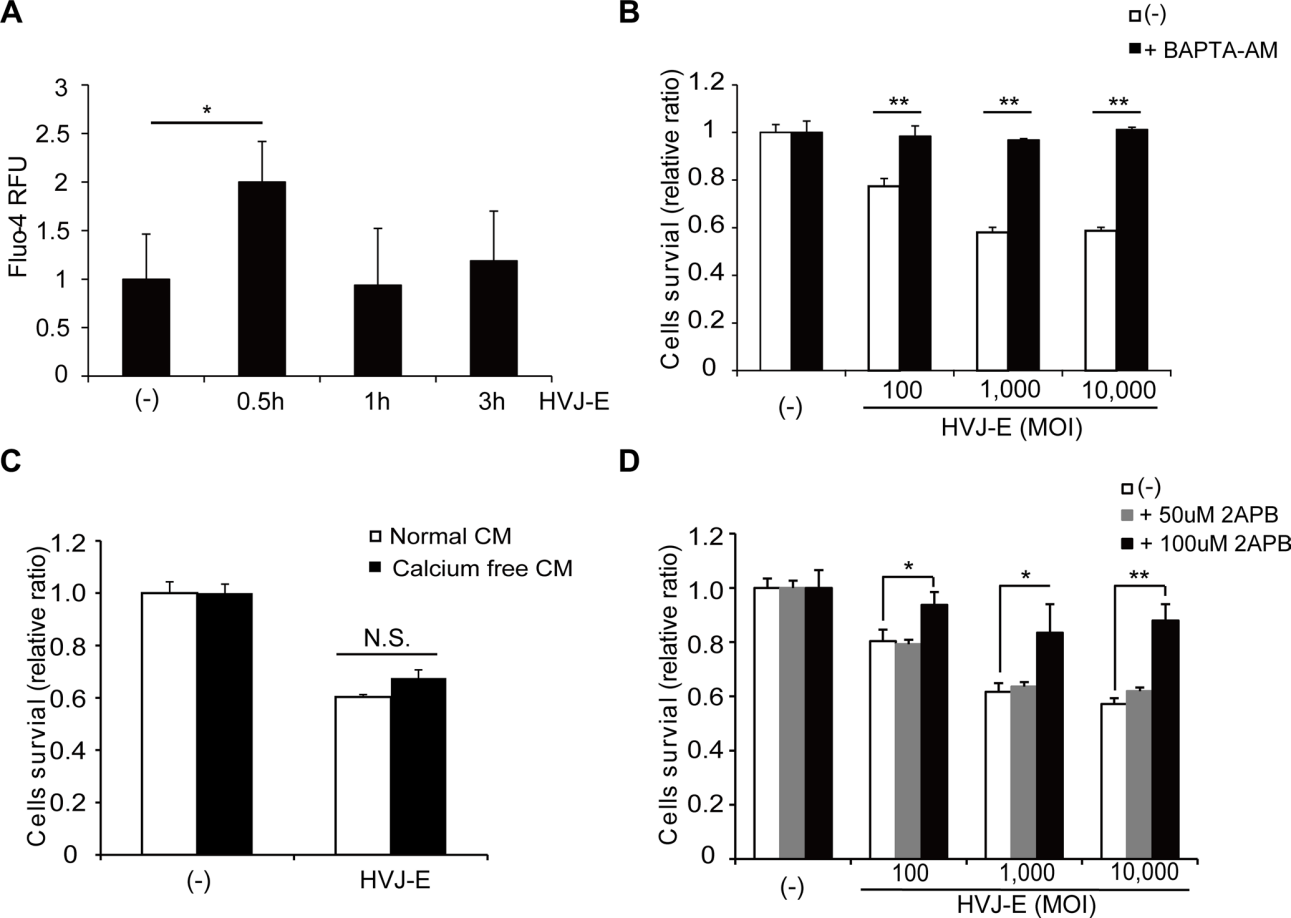
Effects of the increase in cytoplasmic Ca^2+^ on HVJ-E-induced cell death (**A**) Analysis of cytoplasmic Ca^2+^ levels following 0.5, 1 and 3 hours of HVJ-E treatment in NCI-H929 cells using Fluo-4-AM. The data represent the mean ± SD of three independent experiments (*P < 0.05, Tukey-Kramer test). (**B**) NCI-H929 cells were treated with different doses of HVJ-E in the presence or absence of the calcium chelator BAPTA-AM, and cell viabilities were assessed after 48 hours using the MTS assay. The data represent the mean ± SD of three independent experiments (**P < 0.01, Student's unpaired *t*-test). (**C**) Effect of the inhibition of Ca^2+^ influx from the extracellular space on HVJ-E-induced cell death. The MTS assay was performed after HVJ-E treatment for 48 hours in NCI-H929 cells cultured in normal or calcium-free medium. CM: cultured medium. The data represent the mean ± SD of three independent experiments (Tukey–Kramer test, *p* = N.S., no significant difference). (**D**) NCI-H929 cells were treated with different doses of HVJ-E in the presence or absence of the IP3R inhibitor 2-aminoethoxydiphenylborate (2-APB) for 48 hours, and the cell viabilities were examined using the MTS assay. The data represent the mean ± SD of three independent experiments (**P < 0.01, Student's unpaired *t*-test).

Previous studies have shown that HVJ-E-mediated cell death is induced by the recognition of HVJ-E RNA genome fragments with RNA receptor retinoic-acid inducible gene-I (RIG-I) or an elevation of the cytosolic Ca^2+^ concentration [26, 34]. We found that HVJ-E failed to induce RIG-I expression in MM cells (NCI-H929, U266 and MM1S; Supplementary Figure 4A). Moreover, transfection of HVJ-E RNA genome fragments did not inhibit the viability of NCI-H929 cells (Supplementary Figure 4B). These results suggest that RIG-I signaling is not involved in HVJ-E-induced apoptosis in MM cells. Therefore, we next focused on the cytosolic Ca^2+^ concentration of HVJ-E-treated MM cells. NCI-H929 and MM1S cells were exposed to HVJ-E for 0.5, 1 and 3 hours, and cytoplasmic Ca^2+^ levels were measured using Fluo-4-AM. Cytoplasmic Ca^2+^ peaked after 0.5 hours of HVJ-E treatment (Figure 3A, Supplementary Figure 5A). In addition, NCI-H929 and MM1S cells were treated with HVJ-E and a Ca^2+^ chelator (BAPTA-AM) to prevent the effects of Ca^2+^ elevation, and BAPTA-AM almost entirely abrogated HVJ-E-induced cytotoxicity in NCI-H929 cells (Figure 3B, Supplementary Figure 5B).

Next, we investigated the source of the increase in free Ca^2+^ in the cytoplasm of HVJ-E-treated MMs. Although the NCI-H929 cells were cultured in Ca^2+^-free medium to block Ca^2+^ influx from the extracellular space, HVJ-E exhibited cell toxicity in common with the culture in normal medium (Figure 3C). However, HVJ-E-mediated cell death was inhibited in a dose-dependent manner by 2-aminoethoxydiphenyl borate (2-APB), which is an inhibitor of the inositol 1,4,5-trisphosphate receptor (IP3R)/Ca^2+^ channel in the endoplasmic reticulum (ER), (Figure 3D, Supplementary Figure 5C). These results suggested that HVJ-E elevated cytoplasmic Ca^2+^ levels by efflux from the ER but not influx from the extracellular space to induce apoptosis in MM cells.

### Increase of cytosolic calcium by HVJ-E treatment induced apoptosis by suppressing c-Myc expression in MM

**Figure 4:**
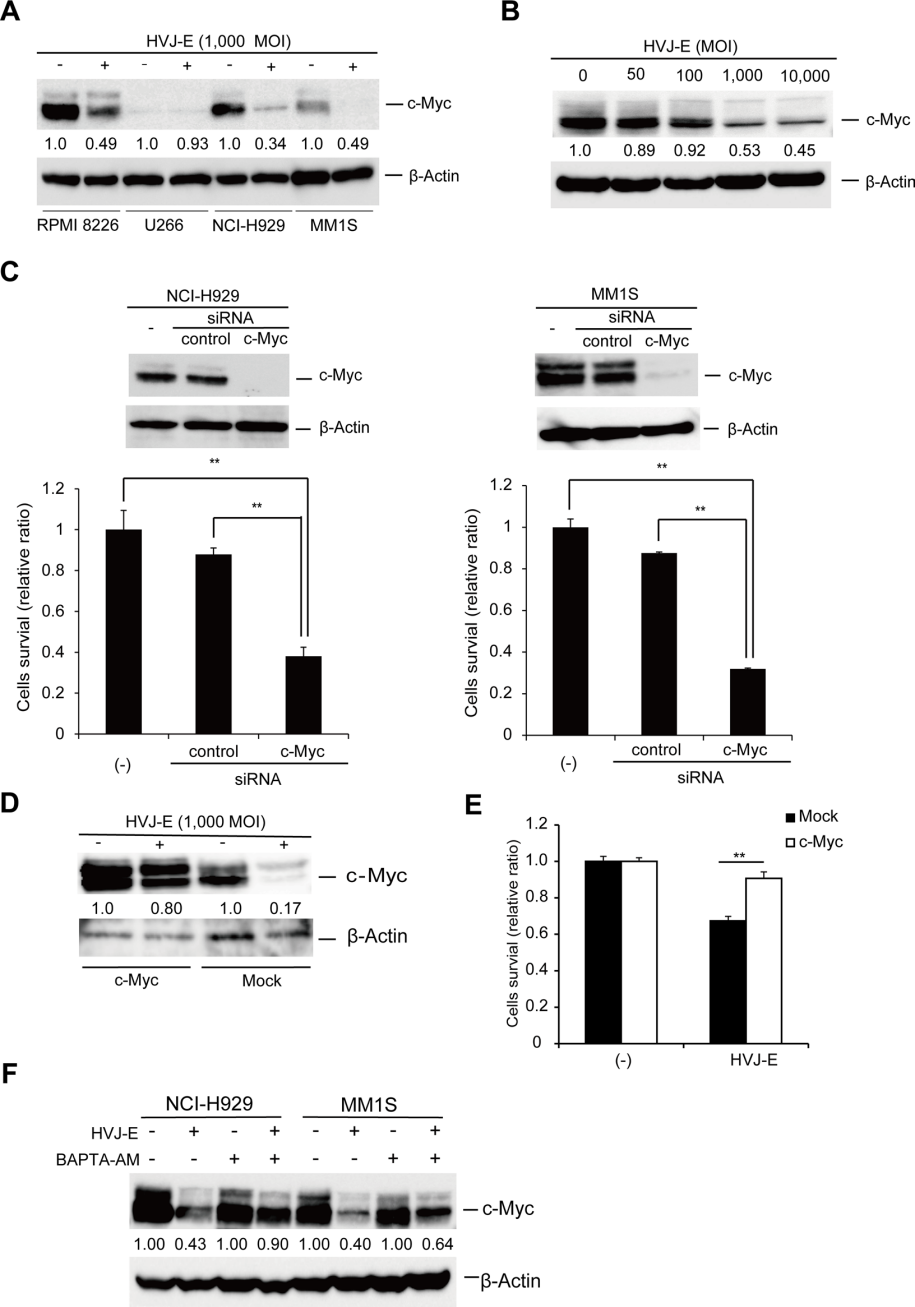
HVJ-E-induced apoptosis is associated with c-Myc downregulation (**A**) Immunoblotting study of c-Myc expression after HVJ-E treatment (1000 MOI, 24 hours) in MM cell lines (RPMI-8226, NCI-H929, U266, and MM1S). (**B**) Dose-dependent effects of HVJ-E treatment on c-Myc expression in NCI-H929. (**C**) c-Myc and expression were evaluated by immunoblotting analysis in untreated, control and c-Myc siRNA-transduced NCI-H929 and MM1S cell lines, Cell viability was measured using the MTS assay at 72 hours after the transduction of control or c-Myc siRNA into NCI-H929 and MM1S cells. The data represent the mean ± SD of three independent experiments (**P < 0.01, Student's unpaired *t*-test). (**D**) Immunoblotting of whole-cell lysates from empty vector- or c-Myc expression vector-transduced MM1S cells after treatment with HVJ-E (1000 MOI, 24 hours). (**E**) Measurement of the cell viability of either empty or c-Myc-overexpressing MM1S cells treated with HVJ-E (1000 MOI, 36 hours) using the MTS assay. The data represent the mean ± SD of three independent experiments (**P < 0.01, Student's unpaired *t*-test). (**F**) Detection of c-Myc expression in NCI-H929 and MM1S cells after treatment with HVJ-E with or without BAPTA-AM for 20 hours by immunoblotting.

In this study, we demonstrated that in contrast to MM1S and NCI-H929 cells, U266 cells showed a lack of response to HVJ-E (Figure 1A, Supplementary Figure 1D). We considered that this differential susceptibility to HVJ-E might result from different expression of genes targeted by HVJ-E, which determine cell fate. Previous studies demonstrated that aberrant c-Myc expression is associated with myeloma cell survival and proliferation [2, 5]. Therefore, we focused on the regulation of c-Myc expression in HVJ-E-treated MM cells. As shown in Figure 4A, c-Myc expression was detected in NCI-H929, MM1S and RPMI-8226 cells, and c-Myc expression was significantly suppressed by HVJ-E treatment. However, c-Myc expression was barely detected in U266 cells, as reported in a previous study [8]. c-Myc expression in NCI-H929 cells was suppressed by HVJ-E in a dose-dependent manner (Figure 4B). To further understand the correlation between c-Myc expression and apoptosis, we knocked down c-Myc expression in NCI-H929 and MM1S cells. c-Myc knockdown led to a decrease in cell viability (Figure 4C) and the cleavage of the apoptosis marker caspase-3 (Supplementary Figure 6). Moreover, c-Myc overexpression in MM1S cells inhibited HVJ-E-mediated c-Myc downregulation and cell death (Figure 4D, 4E). Taken together, these results suggest that HVJ-E-induced c-Myc downregulation causes apoptosis in MM cells, and the endogenous c-Myc expression level determines the susceptibility of MM cell lines to HVJ-E.

To determine whether the increase in cytosolic Ca^2+^ was required for c-Myc downregulation, c-Myc expression was examined in the presence of BAPTA-AM-treated MM cells. NCI-H929 and MM1S cells were treated with 1,000 MOI HVJ-E in the presence or absence of BAPTA-AM, and c-Myc expression was examined by immunoblotting (Figure 4F). HVJ-E alone remarkably decreased c-Myc expression, while BAPTA-AM prevented HVJ-E-induced c-Myc downregulation. These data suggest that the HVJ-E mediated increase in Ca^2+^ in the cytoplasm is an upstream signaling element in the downregulation of c-Myc, which determines HVJ-E-mediated cytotoxicity in MM cells.

**Figure 5:**
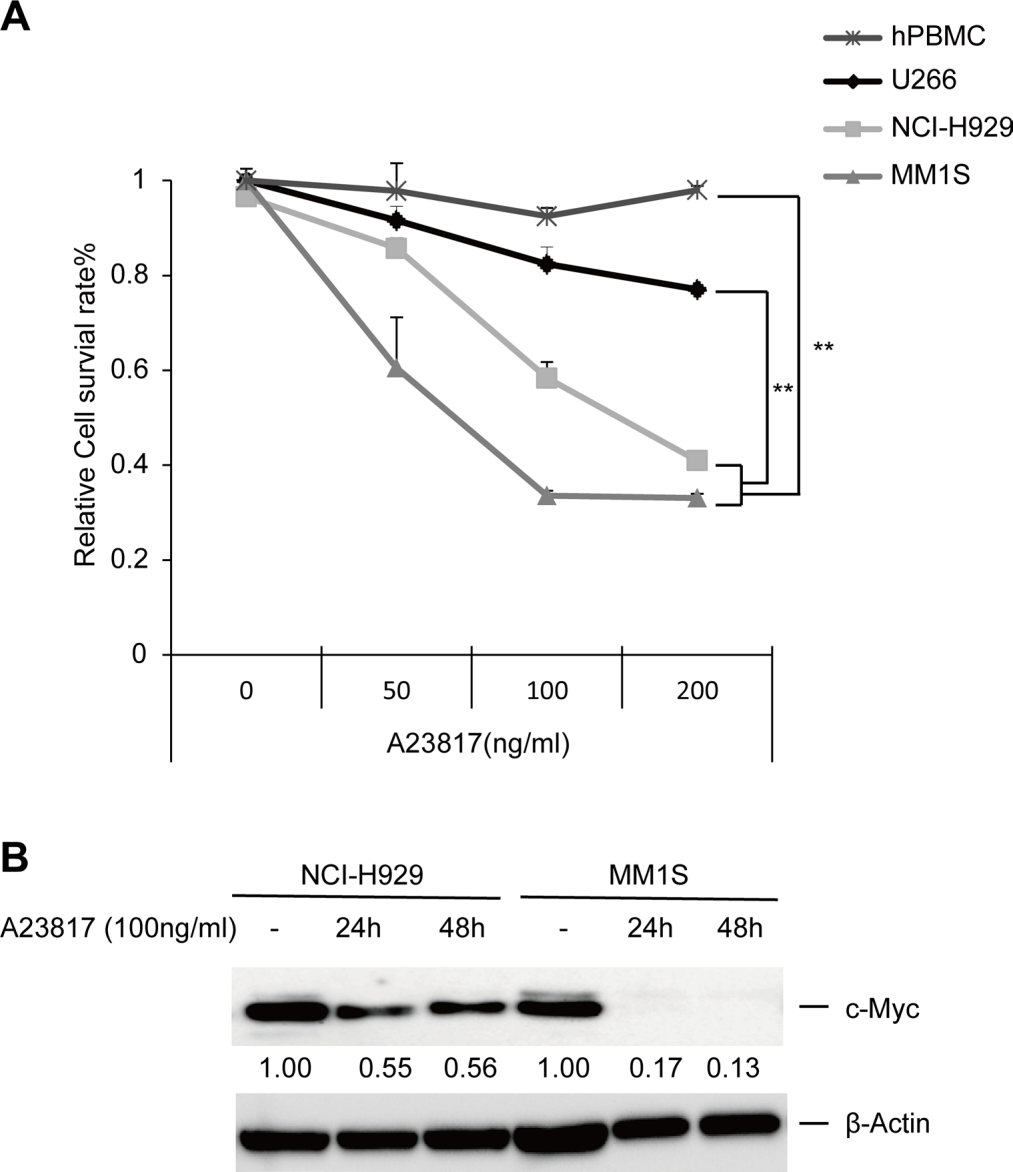
Increasing levels of cytoplasmic Ca^2+^ induce cell death and c-Myc downregulation in MM cells (**A**) MM cell lines (NCI-H929, MM1S and U266) and PBMCs were exposed to different doses of A23817. The relative cell survival ratio was measured after 48 hours using the MTS assay. The data represent the mean ± SD of three independent experiments (**P < 0.01, Tukey–Kramer test). **(B**) Time-dependent effects of A23817 treatment (100 ng/ml) on c-Myc expression in NCI-H929 and MM1S cells, as analyzed by immunoblotting.

We next examined the key factor underlying the induction of HVJ-E-mediated apoptosis via an increase in cytosolic Ca^2+^. We showed that a Ca^2+^ ionophore (A23817) significantly decreased the viability of NCI-H929 and MM1S cells compared to U266 cells and PBMCs (Figure 5A). Meanwhile, in addition, A23817 induced c-Myc downregulation in NCI-H929 and MM1S in a time-dependent manner (Figure 5B). These results confirmed that an increase in cytosolic free Ca^2+^ induced cell death in MMs independently of HVJ-E treatment.

### c-Myc downregulation by calcium signal results from both enhanced degradation of c-Myc protein and suppression of c-Myc transcription

**Figure 6:**
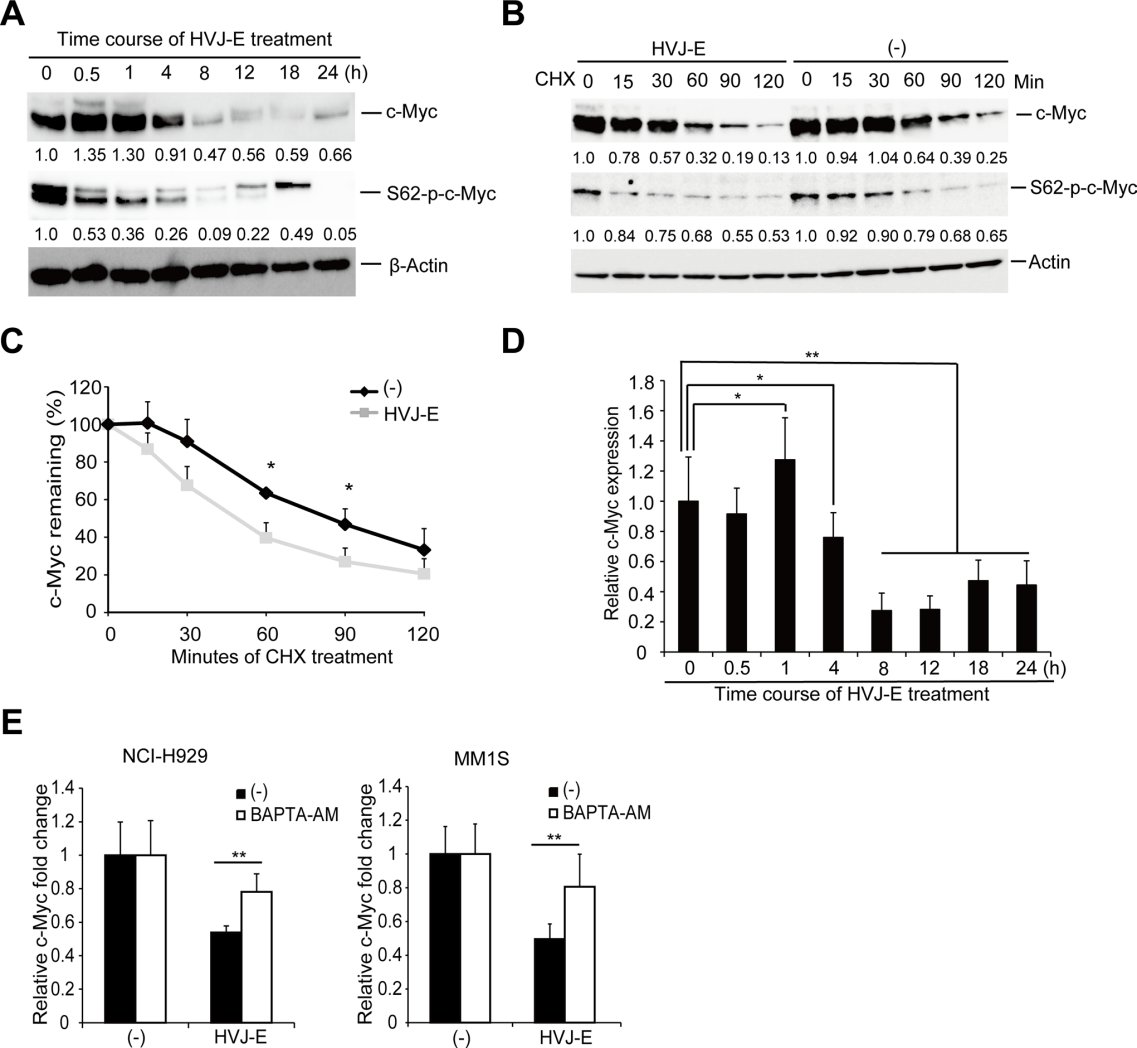
HVJ-E decreases S62-p-c-Myc expression and destabilizes c-Myc protein, BAPTA-AM prevented HVJ-E-induced c-Myc transcription downregulation (**A**) Immunoblotting analyses of the time-dependent effects of HVJ-E treatment on c-Myc and S62-p-c-Myc expression in NCI-H929 cells. (**B**) NCI-H929 cells were treated with cycloheximide (CHX) with or without HVJ-E at the indicated time points, and c-Myc and S62-p-c-Myc expression levels were analyzed by immunoblotting analysis. (**C**) c-Myc levels were quantitated relative to the levels of β-actin in Figure 6B and graphed as the percentage of c-Myc protein remaining after CHX treatment. The data represent the mean ± SD of three independent experiments (*P < 0.05, Student's unpaired *t*-test). (**D**) Quantitative RT-PCR analysis of *c-Myc* levels in HVJ-E-treated NCI-H929 cells (1000 MOI, 0–24 hours). The data are presented as a ratio of *c-Myc* expression at each time point compared with the baseline c-Myc expression (*P < 0.05, **P < 0.01, *t* = 0 h, Tukey–Kramer test). (**E**) Measurement of c-Myc transcriptional expression in NCI-H929 and MM1S cells after treatment with HVJ-E with or without BAPTA-AM for 20 hours by quantitative RT-PCR analysis. The data are presented as a ratio of c-Myc expression compared with baseline c-Myc expression. The data represent the mean ± SD of three independent experiments (**P < 0.01, Student's unpaired *t*-test).

c-Myc expression can be controlled by post-translational and transcriptional regulation [35, 36]. A post-translational pathway that affects c-Myc protein stability is dependent on the phosphorylation of two conserved amino acids: Serine 62 (S62) and Threonine 58 (T58). The elevation of S62 and loss of T58 phosphorylation of c-Myc protein increases Myc protein stability in several solid cancers [37–39]. The decrease in S62 phosphorylation could be a potential approach for reducing the levels of c-Myc protein in cancers. Here, we observed that during the HVJ-E-induced time-dependent downregulation of c-Myc protein, c-Myc S62 phosphorylation decreased at an earlier time point compared with the reduction of c-Myc whole protein and c-Myc T58 phosphorylation in NCI-H929 cells (Figure 6A).

Next, we tested whether HVJ-E would accelerate c-Myc degradation. We cotreated NCI-H929 cells with HVJ-E and cycloheximide (CHX) to inhibit *de novo* protein synthesis, and c-Myc protein levels were measured by immunoblot analysis over 2 hours. As shown in Figure 6B and 6C, HVJ-E treatment significantly increased c-Myc protein degradation. These results indicated that HVJ-E downregulated c-Myc expression by decreasing the S62 phosphorylation of c-Myc protein and accelerating the rate of c-Myc protein degradation.

The HVJ-E-mediated acceleration of c-Myc degradation was significant but slight because the levels of remaining c-Myc protein became nearly equivalent in the presence or absence of HVJ-E treatment after 90 min of CHX treatment (Figure 6B and 6C). Therefore, we hypothesized that other factors might also participate in the HVJ-E-mediated c-Myc downregulation. Next, we focused on c-Myc transcriptional regulation and examined endogenous c-Myc mRNA levels by real-time PCR in HVJ-E-treated NCI-H929 cells. As shown in Figure 6D, HVJ-E induced a transient increase in c-Myc mRNA within one hour and a decrease to below the baseline level after four hours of treatment. Moreover, BAPTA-AM inhibited the HVJ-E-induced suppression of c-Myc transcription in NCI-H929 and MM1S cells (Figure 6E). Taken together, these results suggest that the HVJ-E-induced increase in cytoplasmic Ca^2+^ mediated the downregulation of c-Myc expression mainly by suppressing c-Myc transcription.

### Calcium-mediated suppression of c-Myc transcription depends on SMADs activation

**Figure 7:**
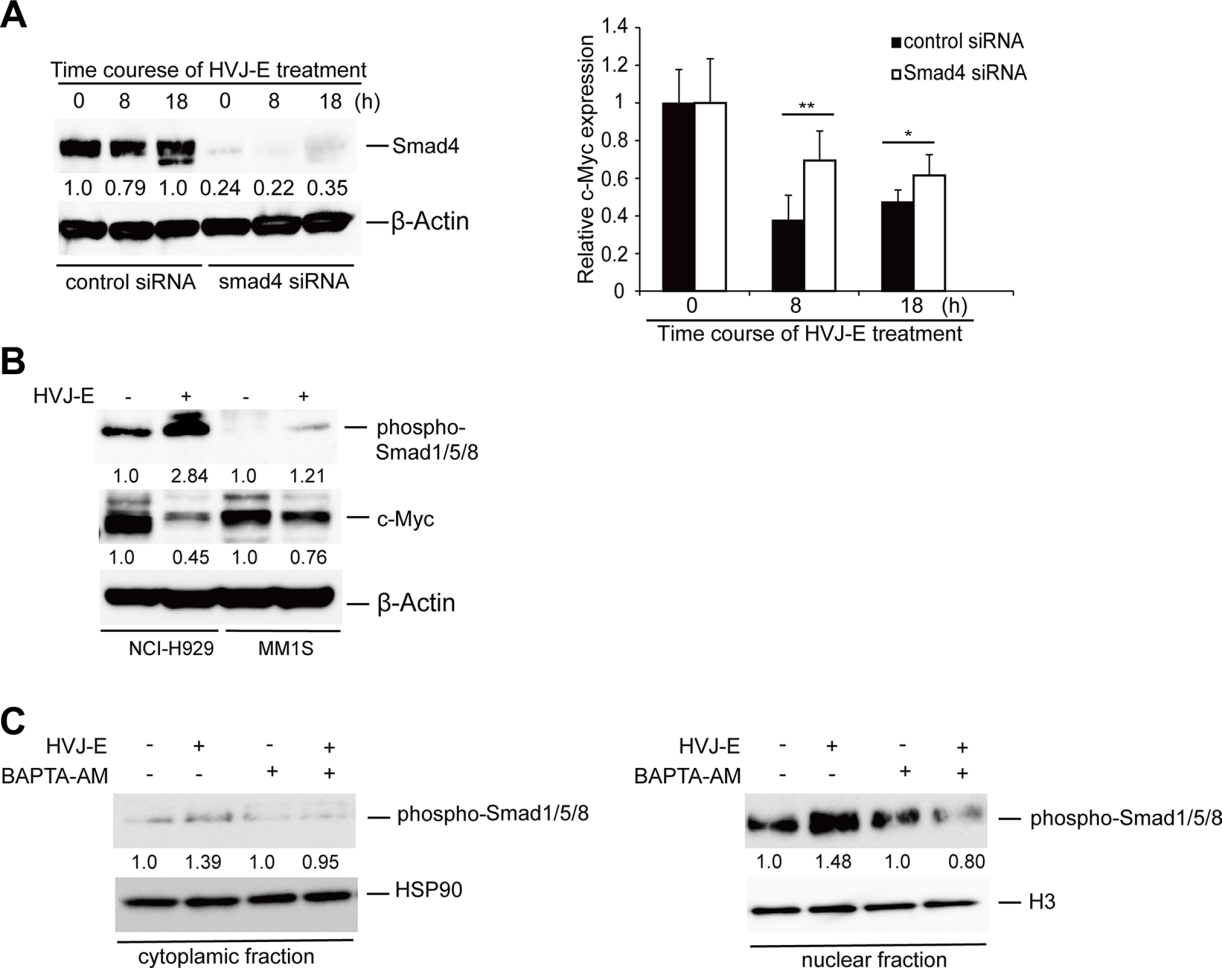
The involvement of SMADs in HVJ-E-induced suppression of c-Myc transcription (**A**) Left panel: Time-dependent Smad4 expression was measured in control siRNA- and Smad4 siRNA-transduced NCI-HJ929 cells by immunoblotting analysis. Right panel: Time-dependent effect of HVJ-E on c-Myc mRNA expression in control siRNA- and Smad4 siRNA-transduced NCI-H929 cells by quantitative RT-PCR analysis. The data are presented as the ratio of c-Myc expression compared with baseline c-Myc expression. The data represent the mean ± SD of three independent experiments (*P < 0.05, **P < 0.01, Student's unpaired *t*-test). (**B**) Protein levels of phospho-SMAD1/5/8 and c-Myc were analyzed in the MM cell lines NCI-H929 and MM1S with or without HVJ-E treatment (1000 MOI, 8 hours). (**C**) Measurement of the cytoplasmic and nuclear fraction of activated SMAD1/5/8 in NCI-H929 cells after treatment with HVJ-E with or without BAPTA-AM for 8 hours by immunoblotting.

Finally, we investigated the mechanism by which c-Myc transcription was inhibited by the cytoplasmic Ca^2+^ increase in MMs. A previous study demonstrated that the cytosolic increase in Ca^2+^ induces Smad1 phosphorylation to promote Smad1 nuclear translocation [40]. In addition, either SMAD1 or SMAD1/5/8 regulates c-Myc transcription by binding to SMAD4 to form a Smad complex [41–43]. As shown in Figure 7A, the transient knockdown of Smad4 in NCI-H929 cells by siRNA inhibited the HVJ-E-induced suppression of c-Myc transcription. HVJ-E induced the phosphorylation of Smad1/5/8 as well as the repression of c-Myc in NCI-H929 and MM1S cells (Figure 7B). Moreover, in HVJ-E-treated NCI-H929 cells, the immunoblotting results clearly revealed a significant increase in phosphorylated SMAD1/5/8 in the nuclear fraction following HVJ-E stimulation, and BAPTA-AM abolished the HVJ-E-mediated phosphorylation and nuclear translocation of SMAD1/5/8 (Figure 7C, Supplementary Figure 7). Taken together, Figure 7 suggests that the increase in cytosolic Ca^2+^mediating Smad activation is involved in the downregulation of HVJ-E-induced c-Myc transcription in MM.

## DISCUSSION

In the present study, we demonstrated that HVJ-E effectively induced apoptosis in various MM cells by downregulating c-Myc via an elevation of cytoplasmic Ca^2+^ levels. Calcium-induced c-Myc downregulation resulted from both suppression of c-Myc transcription via activation of SMAD1/5/8 and acceleration of c-Myc protein degradation. MM cells (NCI-H929, MM1S, and RPMI8226) which were highly expressing c-Myc were preferentially killed by increase of calcium signal via fusion with HVJ-E, but U266 MM cell line and PBMCs were not killed. Therefore, MM cells with c-Myc addiction can be killed by HVJ-E via elevation of cytosolic calcium. c-Myc is an important transcription factor for the regulation of cancer cell proliferation and highly controls malignant state in various cancer cells [44, 45]. In previous reports, even transient inactivation of c-Myc leads to tumor collapse in mice models [46–48]. Therefore, there is a possibility that HVJ-E is able to induce cell death in malignant cancer cells with c-Myc addiction via the increase of cytoplasmic Ca^2+^ without affecting normal cells lacking c-Myc addiction. Moreover, as shown in this report, besides inhibiting c-Myc transcription, developing methods that accelerate the rate of c-Myc destruction by destabilizing c-Myc protein may ultimately prove effective in tumor settings [49, 50].

It is well known that Ca^2+^ level modification plays important roles in regulating cell death (for example, apoptosis [51, 52] and autophagy [53]). However, in this study, we demonstrated that HVJ-E induced Ca^2+^-dependent apoptosis, but autophagy was not involved in HVJ-E-induced cell death (unpublished data). Numerous Ca^2+^-dependent downstream effectors can efficiently transduce extracellular signals into apoptosis [52, 54]. However, protein kinase C (PKC) [55], calpains activation and ER stress [54] were not the downstream events involved in Ca^2+^ level modification-induced apoptosis by HVJ-E (unpublished data). Several papers suggested the relationship between the increase in cytosolic Ca^2+^ and decrease in c-Myc transcription. The Ca^2+^ ionophore A23817 downregulates c-Myc expression in human promyelocytic leukemia and T lymphoblastic leukemia cells (HL-60, MOLT-4) by inhibiting RNA elongation [56, 57]. However, those reports showed no evidence that the downregulation of c-Myc is responsible for A23817-induced cell death by increasing cytosolic Ca^2+^levels. The crosslinking of IgM in murine B-lymphoma cells induces G1 arrest and apoptosis by p27^Kip1^ accumulation, which results from either c-Myc downregulation or increase of cytosolic calcium [58]. This report indicates that c-Myc downregulation and cytosolic calcium increase are independently involved in apoptosis of lymphoma cells. No relationship between two pathways is clearly shown. Thus, till now, there have been no reports putting links among cytosolic Ca^2+^ increase, c-Myc downregulation and apoptosis.

In the present study, we found that HVJ-E-induced cell death was dependent on the downregulation of c-Myc (Figure 4E) and that the intracellular Ca^2+^ chelator BAPTA-AM prevented the HVJ-E-induced c-Myc downregulation (Figure 4F and Figure 6E) and cell death of MM cells (Figure 3B). Taken together, our results confirm that the accumulation of cytosolic Ca^2+^ upstream of c-Myc downregulation leads to apoptosis in HVJ-E-treated MMs. In this study, we also found that HVJ-E treatment increased the expression of the pro-apoptotic protein Bim in MM cells (Supplementary Figure 3B, 3C). The relationship between c-Myc and Bim regulation was well documented in MM cells in a previous study [59]. Namely, shRNA-mediated c-Myc suppression subsequently increases Bim expression in myeloma cells, which indicates that the silencing of c-Myc induces myeloma cell apoptosis [59]. In our study, HVJ-E mediated both c-Myc downregulation and Bim upregulation, and the siRNA-mediated downregulation of Bim inhibited the cytotoxicity of HVJ-E in MM cells (Supplementary Figure 3D). These results suggest that HVJ-E-induced c-Myc downregulation likely results in Bim upregulation, which leads to apoptosis in MM cells.

HN glycoprotein enables HVJ-E particles to adhere to the host cell surface [60]. A previous study indicated that HVJ-E-mediated cytotoxicity is dependent on the expression of GD1a and SPG, which are HN receptors [61], on target cells. In the present study, we showed that HVJ-E exhibited cytotoxic activity against MM cells, although, MM cells had no conventional receptors for acidic gangliosides recognized by HN glycoprotein of HVJ (Supplementary Figure 8A); however, HVJ-E could still bind to MM cells (Supplementary Figure 8B). Moreover, HN-depleted HVJ-E could adhere to the cell surface of NCI-H929 cells (Supplementary Figure 9A) and induce apoptosis via membrane fusion (Supplementary Figure 9B and 9C). For these reasons, we suggest that MMs may express an unknown receptor for F binding to induce membrane fusion. MM is a systemic cancer, and systemic treatment is required for MM therapy. Because HN is responsible for not only HVJ-E particle-adhesion but also hemagglutination, systemic administration of HVJ-E is not practical. Although HN depletion of HVJ-E decreases both hemagglutinating activity and cell adhesion ability [62], HN-depleted HVJ-E displays added cancer cell affinity and utility for cancer therapy [63]. Here, we showed that MMs had an affinity for HN-depleted HVJ-E. Therefore, the systemic administration of HN-depleted HVJ-E will be a promising tool for MM therapy.

## MATERIALS AND METHODS

### Cell culture

Human MM cell lines (NCI-H929, MM1S, U266, and RPMI-8226) were obtained from the American Type Culture Collection (ATCC; VA, USA). MM1S, U266, and RPMI-8226 cells were cultured in RPMI 1640 medium (Nacalai Tesque, Inc., Kyoto, Japan) containing 10% fetal bovine serum (FBS), 100 U/ml of penicillin, and 100 μg/ml of streptomycin (Nacalai Tesque). NCI-929 cells were cultured in streptomycin and 2mercaptoethanol (Nacalai Tesque) at a final concentration of 0.05 mM. PBMCs were obtained from healthy donors after obtaining informed consent. Five milliliters of whole blood were collected from three healthy donors, and PBMCs were isolated from the blood by cell density centrifugation over a Ficoll-Paque density gradient. PBMCs were washed three times with sterile PBS (Nacalai Tesque). PBMCs were cultured in Dulbecco's modified Eagle's medium (DMEM) and RPMI 1640 mixed 1:1 and supplemented with 100 U/ml of penicillin, 100 μg/ml of streptomycin, 5% heat-inactivated AB human serum (Sigma-Aldrich, Inc., Tokyo, Japan), 10 mM HEPES, and 60 μg/ml of arginine-HCl.

### Preparation of HVJ-E

HVJ (VR-105 parainfluenza 1 Sendai/52, Z strain from the ATCC) was amplified in the chorioallantoic fluid of 14-day-old chick eggs, purified by centrifugation and inactivated by UV irradiation (99 millijoules/cm^2^). The inactivated virus lost the ability to replicate and synthesize viral proteins but retained its membrane fusion activity.

### Chemical treatments

To inhibit caspase activity, 100 μmol/L of the pan-caspase inhibitor Z-VAD-FMK (Medical and Biological Laboratories, Inc., Nagoya, Japan) was added to the cells one hour prior to HVJ-E treatment. Calcium-free conditions were prepared using calcium-free RPMI 1640 medium (US Biological, Inc., MO, USA), and in the cytoplasmic Ca^2+^ inhibition experiments, 5 μmol/L BAPTA-AM or 50 μmol/L 2-aminoethoxydiphenyl borate (2-APB) (Sigma-Aldrich) was added to the cells 1 hour before exposure to HVJ-E. To increase cytoplasmic Ca^2+^, A23817 was used which was purchased from Sigma-Aldrich (Tokyo, Japan).

### Cytotoxicity assays

Cell viability was determined using the Cell Titer 96 Aqueous One Solution Cell Proliferation Assay kit (Promega, Inc., WI, USA). Briefly, after treatment with the compounds at the indicated concentrations for the indicated durations, 100 μl of Cell Titer 96 Aqueous One Solution reagent was added to each well, and the plates were incubated at 37°C in 5% carbon dioxide for 2 hours. After transferring 100 μl of incubation medium from each well into a new 96-well plate, the absorbance at 490 nm was measured.

### Assessment of apoptosis

Apoptosis was evaluated using a PE Annexin V Apoptosis Detection Kit (BD Biosciences, Inc., CA, USA). Briefly, 1 × 10^5^ cells were resuspended in 500 μl of 1× binding buffer, to which was added 5 μl of Annexin V and 5 μl of propidium iodide. The cells were incubated in the dark for 15 minutes. For the flow cytometry analysis, the percentages of the positive cells in the upper right (Annexin V^+^/PI^+^: late apoptotic cells) and lower right quadrants (Annexin V^+^/PI^−^: early apoptotic cells) were summed to obtain the total number of apoptotic cells.

### Measurement of cytoplasmic Ca^2^^+^ in HVJ-E-treated MM cells

The level of intracellular cytosolic calcium was measured using the Fluorescent Indicator Fluo-4 direct calcium assay kit (Invitrogen). NCI-H929 cells were stimulated in the presence of HVJ-E at an MOI of 1,000 for 0, 0.5, 1 and 3 hours. After the incubation, the cells were stained with Fluo-4 according to the manufacturer's instructions. The fluorescence was measured using Infinite M200 at an excitation of 494 nm and emission of 516 nm.

### siRNA and plasmid transfection

Transfection was performed by electroporation using three 10-second pulses at a voltage of 1300 with the Neon^®^ transfection system (Life Technologies) according to the manufacturer's instructions. For c-Myc and Smad4 transient silencing, NCI-H929 and MM1S cells were transfected with SMARTpool Accell human c-Myc siRNA (E-003282-00-0005; Thermo Fisher Scientific, Inc., UT, USA) or Smad4 siRNA (E-003902-00-0005; Thermo Fisher Scientific), and Accell Green Non-targeting siRNA (D-001950-01-20; Thermo Fisher Scientific) was used as a control. We used 100 pmol of siRNA/2.5 × 10^5^ cells. For c-Myc overexpression, plasmid vectors containing a cytomegalovirus (CMV) promoter with or without (control) human c-Myc cDNA were purchased from Thermo Fisher Scientific. One microgram of plasmid per 2.5 × 10^5^ cells was used.

### Real-time quantitative RT-PCR

RNA was extracted from cultured cells using the RNeasy Mini Kit (Qiagen, Tokyo, Japan), and 500 ng of total RNA were converted into cDNA using the High Capacity cDNA Reverse Transcription Kit (Applied Biosystems, Tokyo, Japan). Human c-Myc and β-actin were amplified using SYBR Premix Ex Taq (Takara Bio, Shiga, Japan). All procedures were performed according to the manufacturer's instructions. The following primers were used:

Human β-actin-forward, 5′-GAGCTACGAGCTG CCTGACG-3′, and

Human β-actin-reverse, 5′-GTAGTTTCGTGGAT GCCACAG-3′; and

Human c-Myc forward, 5′-GACCAG- CTGGAGAT GGTGAC-3′, and

Human c-Myc reverse, 5′-GGTCGCAGATGAAA CTCTGG-3′.

### Antibodies and western blotting analysis

Antibodies were obtained from the following commercial sources: anti-β-actin antibody (AC-15, A5441) was purchased from Sigma-Aldrich; the anti-caspase-3 (#9662), anti-caspase-9 (#9502), anti-caspase-8 (#9746), anti-PARP (#9542), anti-c-Myc (#5605), anti-HSP90 (#4874), anti-Smad4 (#9515) and anti-H3 (#9715) antibodies were purchased from Cell Signaling Technology (Tokyo, Japan); the anti-phospho-Smad1/Smad5/Smad8 antibodies recognizing phosphorylated Ser463/465 were purchased from Millipore (MA, USA); and the anti-phospho-Ser62-c-Myc (ab51156) and anti-phospho-Thr58-c-Myc (ab135558) antibodies were purchased from Abcam (Cambridge, MA). After treatment, the cells were washed once with cold PBS, the cell pellets were treated with RIPA buffer, and the total protein was isolated. Protein (10 μg) was resolved in polyacrylamide gels and transferred onto polyvinylidene difluoride (PVDF) membranes (Millipore). The membranes were probed with the primary antibody described above followed by a horseradish peroxidase-linked secondary antibody (GE Healthcare Japan). The signals were detected using Chemi-Lumi One (Nacalai Tesque) with Image Quant LAS 4000 mini software (GE Healthcare, Buckinghamshire, UK). The band density was analyzed using the NIH ImageJ software.

### Measurement of c-Myc protein stability

NCI-H929 cells were treated with 100 μg/ml of CHX in the presence or absence of HVJ-E. Cell lysates were prepared after the indicated time periods from 0–120 minutes. The samples were then immunoblotted to visualize total endogenous c-Myc, S62-c-Myc and β-actin. Protein levels of c-Myc, S62-c-Myc and β-actin were then quantified using Image Quant TL image analysis software (GE Healthcare). The levels of total c-Myc protein and S62-c-Myc (normalized to β-actin) in CHX-treated cells with or without HVJ-E was determined using the value for CHX-untreated cells at time 0 hours as equal to 1. Relative c-Myc levels were then visualized on an X-Y scatter graph plotted using the best-fit exponential curve for the indicated time points.

### Human MM xenograft models

Six- to eight-week old NOG (NOD/Shi-scid, IL- 2Rγ KO) mice were purchased from Clea Japan (Tokyo, Japan) and maintained in a temperature-controlled pathogen-free room. All animal experiments were carried out according to the guidelines of the Institute for Animal Studies at Osaka University (Osaka, Japan). MM1S or NCI-H929 cells (5.0 × 10^6^) in 100 μl of PBS were inoculated subcutaneously into the right flanks of NOG mice (female, 8 weeks old). When the tumors were measurable (approximately 150–200 mm^3^), approximately 10– 14 weeks after MM cell injection, the mice were treated intratumorally with vehicle alone (PBS) or 5,000 HAU of HVJ-E 6 times every two days.

### Statistical analysis

The results are reported as the mean ± standard deviation (SD). The statistical significance of differences was evaluated using the Tukey–Kramer test or Student's unpaired *t*-test, and a *P* value less than 0.05 was considered to be significant.

## SUPPLEMENTARY MATERIALS AND FIGURES


