# Supplementary material for: Cytoplasmic calcium increase via fusion with inactivated Sendai virus induces apoptosis in human multiple myeloma cells by downregulation of c-Myc oncogene
Source: Oncotarget. 2016 Apr 29;7(24):36034–48. doi: 10.18632/oncotarget.9105 (PMC5094981; doi:10.18632/oncotarget.9105)
Supplement: Supplementary file 1 [file oncotarget-07-36034-s001.pdf]

Cytoplasmic calcium increase via fusion with inactivated Sendai virus induces apoptosis in human multiple myeloma cells by downregulation of c-Myc oncogene

Supplementary Materials

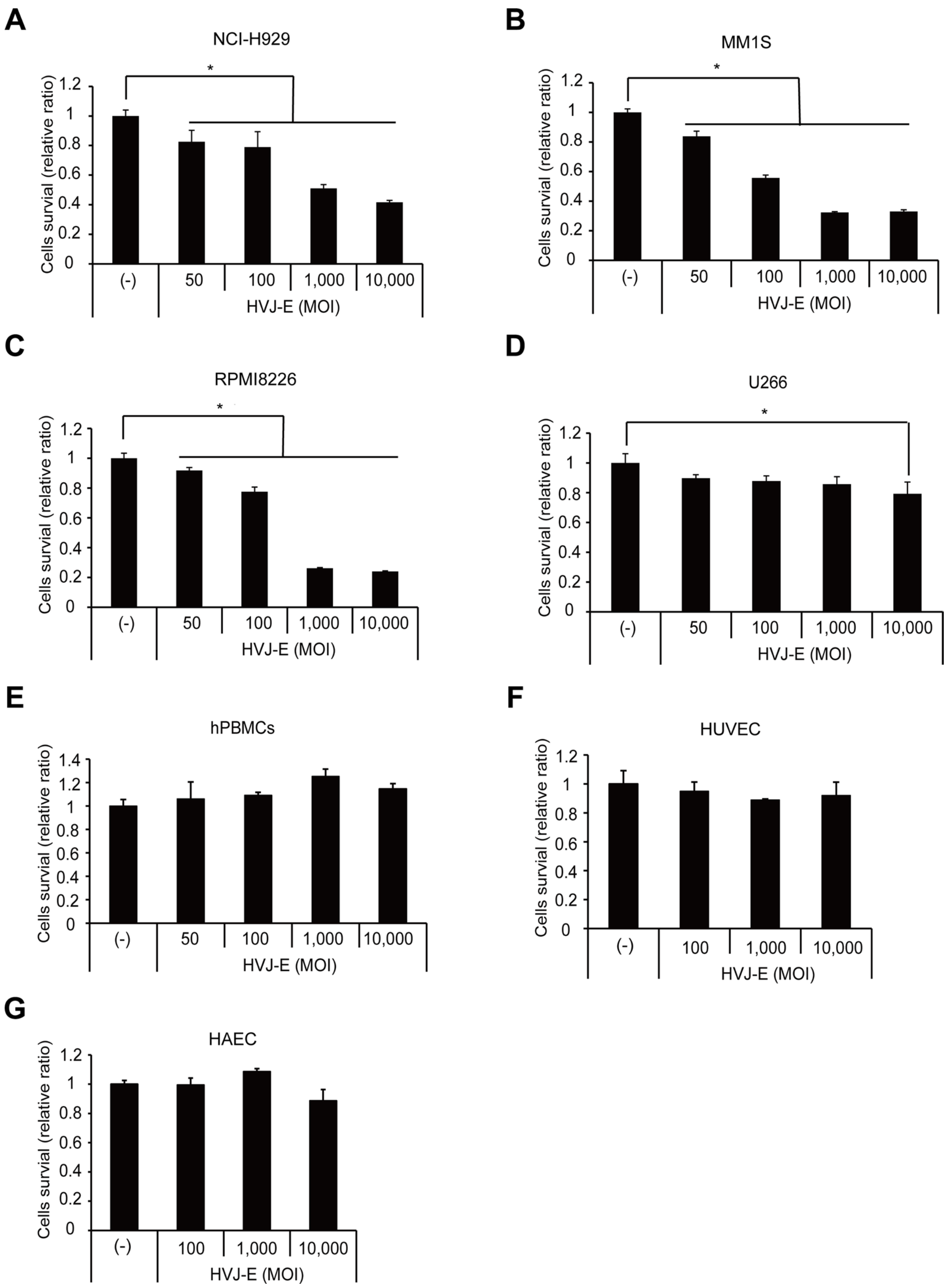

Supplementary Figure S1: HVJ-E induces cell death in MM cells but not in PBMCs and human endothelial cell lines. (**A–G**) MM cell lines (RPMI-8226, MM1S, NCI-H929, and U266), PBMCs from healthy donors and endothelial cell lines: HUVEC and HAEC were treated with several doses of HVJ-E for 72 hours or 48 hours. The viability of the cells was measured using the MTS assay. The data represent the mean ± SD of three independent experiments (*$P < 0.05$, Tukey–Kramer test).

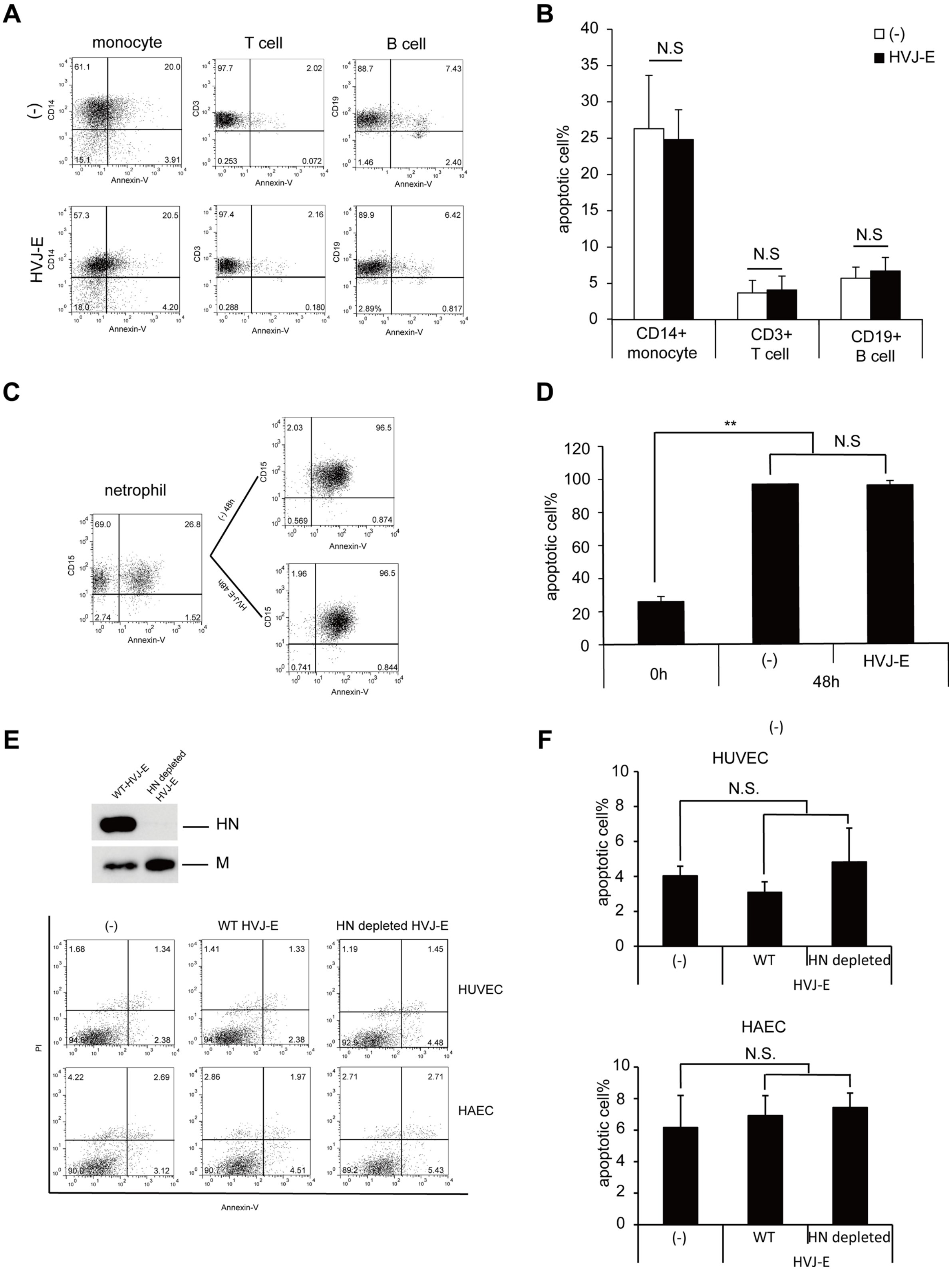

Supplementary Figure S2: HVJ-E did not induce apoptosis in leukocytes from healthy donors and human endothelial cell lines. (**A** and **C**) Apoptotic cells were measured in HVJ-E-treated $CD3^+$ T cells, $CD19^+$ B cells, and $CD14^+$ monocytes from PBMCs and $CD15^+$ neutrophils from healthy donors at a multiplicity of infection (MOI) of 1000 for 48 hours using PI (vertical) and Annexin V (horizontal) double staining analysis by FACS. (**B** and **D**) Quantification plot of the ratio of the apoptotic (Annexin V^+) cells shown in Supplementary Figure 1A and 1C. (**E**) Upper-panel: immunoblotting analysis of HN and M expression in wild-type (WT) HVJ-E and HN-depleted HVJ-E. Lower-panel: apoptotic cells were measured in HUVECs and HAECs after 48 hours of treatment with WT or HN- depleted HVJ-E (1,000 MOI) by FACS. (**F**) Quantification plot of the ratio of apoptotic (Annexin V^+) cells shown in E. The data represent the mean ± SD of three independent experiments (*P* = N.S, no significant difference, Tukey–Kramer test).

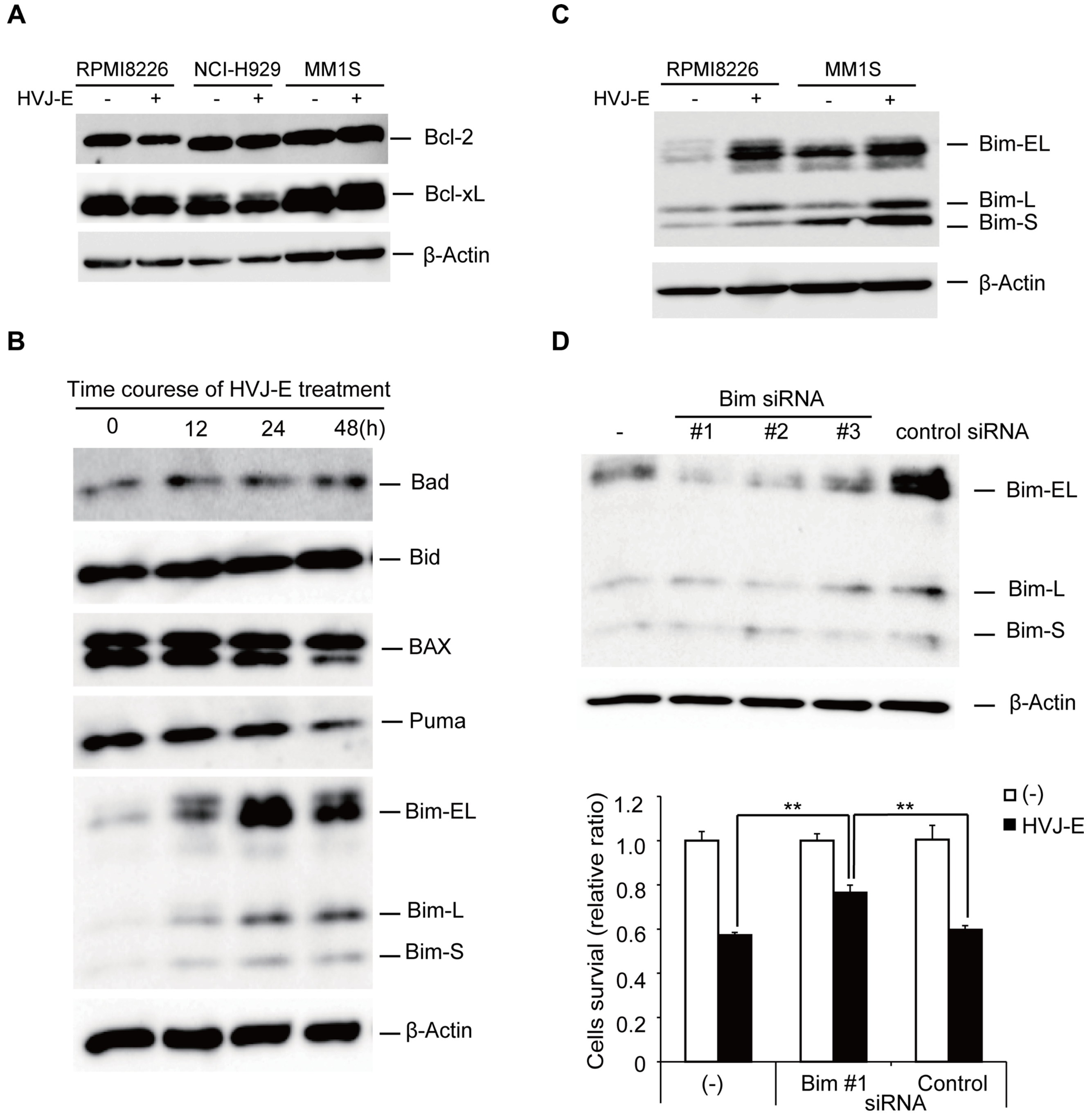

Supplementary Figure S3: Bim upregulation is involved in HVJ-E-induced apoptosis in MM cells. (**A**) Immunoblotting analysis of the expression of the anti-apoptotic proteins Bcl-2 and Bcl-xL in RPMI-8226, NCI-H929 and MM1S cells (1000 MOI, 24 hours) (**B**) Immunoblotting of the expression of proapoptotic proteins (Bax, Bid, Bad, Puma and Bim) in HVJ-E-treated NCI-H929 cells (1,000 MOI, 0-24 hours). (**C**) Immunoblotting analysis of the effect of HVJ-E on Bim expression in MM1S and RPMI-8226 cells (1000 MOI, 20 hours). (**D**) Immunoblotting of Bim expression in NCI-H929 cells after transduction with Bim siRNA (#1, #2, and #3) or control siRNA for 24 hours, and the cell viability of either control or # 1 Bim siRNA-transfected NCI-H929 cells treated with HVJ-E by the MTS assay (1000 MOI, 48 hours). The data represent the mean ± SD of three independent experiments (**$P < 0.01$, Tukey–Kramer test).

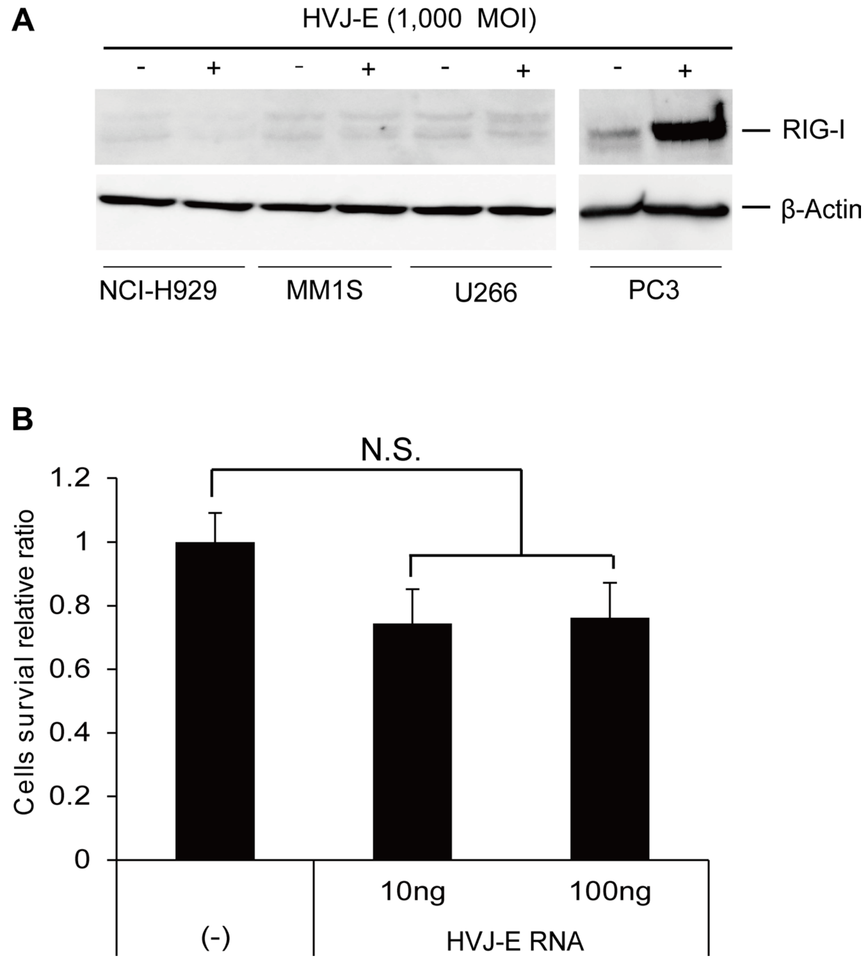

Supplementary Figure S4: The RIG-I signaling pathway is not involved in HVJ-E-mediated cell death. (**A**) The expression level of RIG-I was measured in MM cell lines (NCI-H929, MM1S and U266) and the PC3 cell line which with or without HVJ-E (1000 MOI, 24 hours). (**B**) The relative cell survival ratio was detected in NCI-H929 cells at 72 hours following the transfer of different doses of HVJ-E RNA fragments. The data represent the mean ± SD of three independent experiments (*P* = N.S., no significant difference, Tukey–Kramer test).

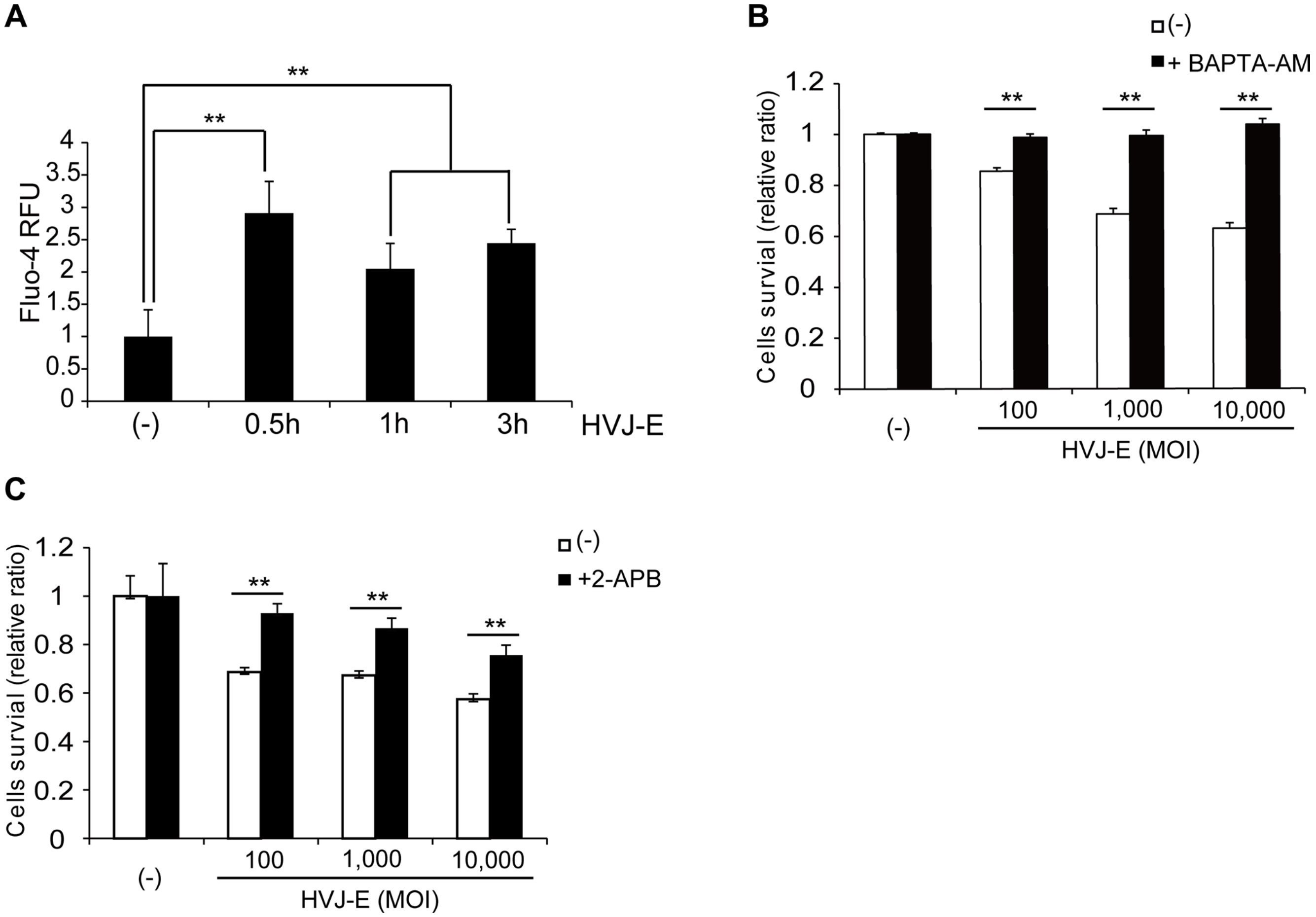

Supplementary Figure S5: Effects of increase in cytoplasmic Ca^{2+} on HVJ-E-induced cell death in MM1S cells. (**A**) Analysis of cytoplasmic Ca2+ levels following 0.5, 1 and 3 hours of HVJ-E treatment in MM1S cells using Fluo-4-AM (**$p < 0.01$, Tukey-Kramer test). (**B**) MM1S cells were treated with different doses of HVJ-E in the presence or absence of the calcium chelator BAPTA-AM, and cell viabilities were assessed after 48 hours using the MTS assay. The data represent the mean ± SD of three independent experiments (**$P < 0.01$, Student's unpaired *t*-test). (**C**) MM1S cells were treated with different doses of HVJ-E in the presence or absence of the IP3R inhibitor 2-aminoethoxydiphenylborate (2-APB) for 48 hours, and cell viabilities were examined using the MTS assay. The data represent the mean ± SD of three independent experiments (**$P < 0.01$, Student's unpaired *t*-test).

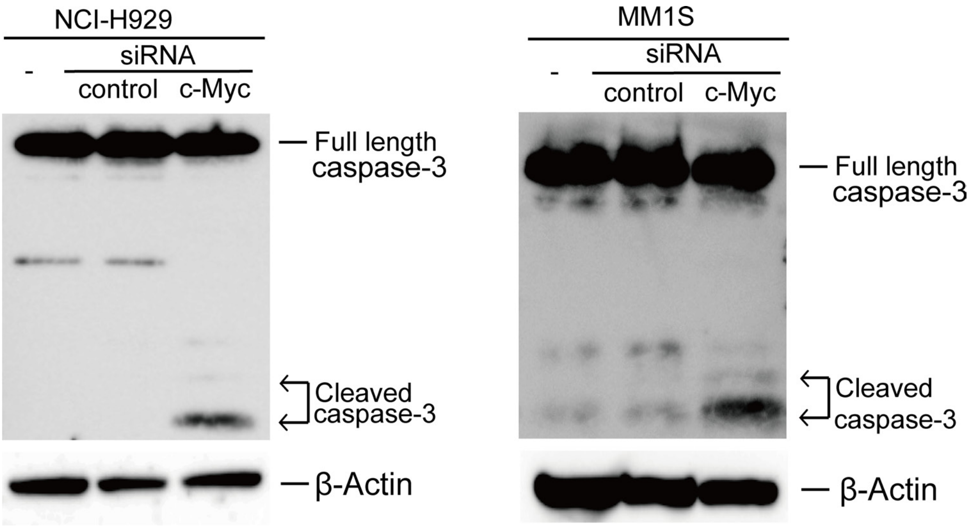

Supplementary Figure S6: Knockdown of c-Myc expression in MM cells induces caspase-3 cleavage. Caspase-3 cleavage was detected by immunoblotting analysis in NCI-H929 and MM1S cell lines 48 hours after the transfer of no, control or c-Myc siRNA.

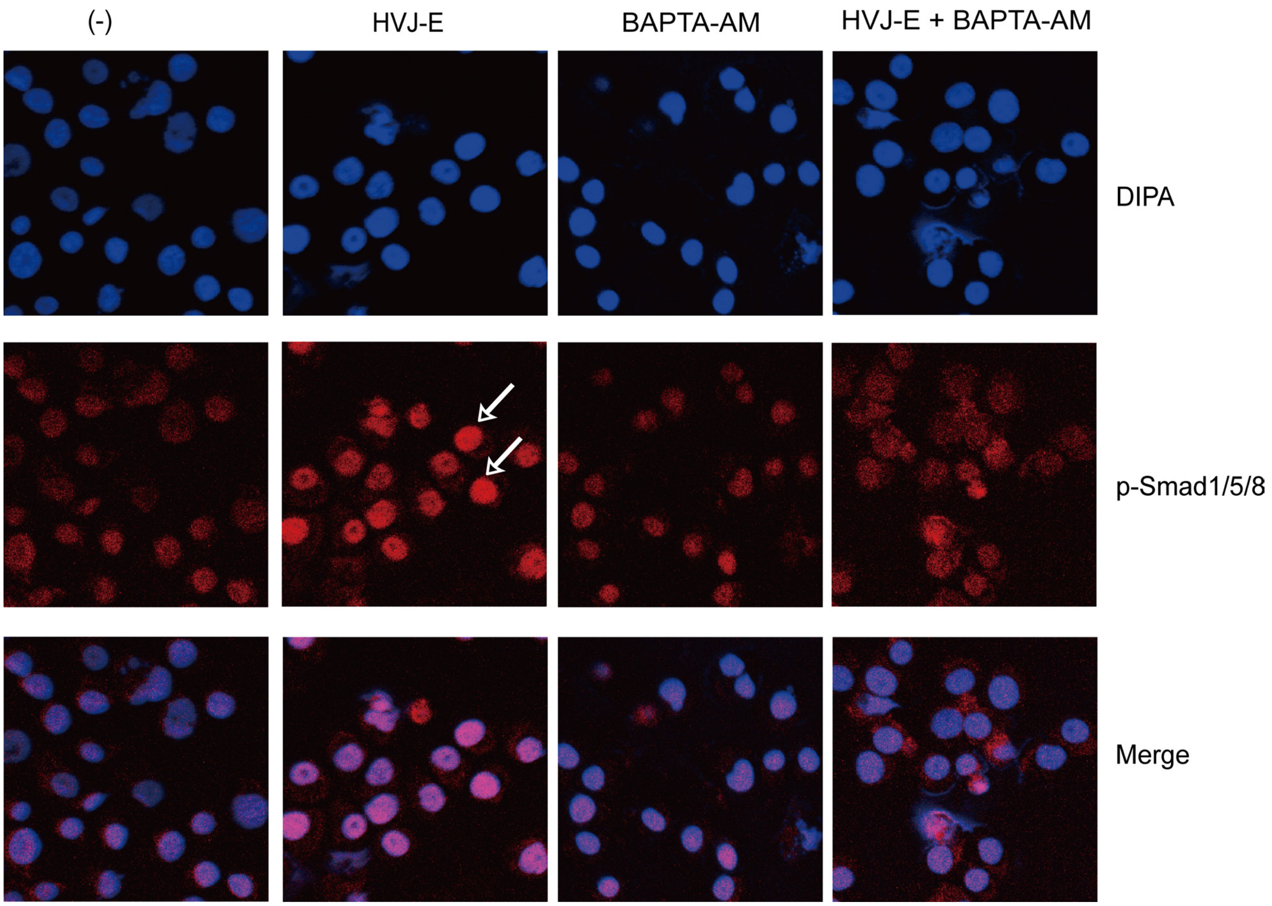

Supplementary Figure S7: BAPTA-AM prevented HVJ-E-induced SMAD1/5/8 phosphorylation and nuclear translocation. The expression of phosphorylated SMAD1/5/8 (red) was visualized in NCI-H929 after treatment with HVJ-E with or without BAPTA-AM for 8 hours by immunofluorescence. The nuclei were stained with DAPI (blue). The arrows indicate nuclear localization of phosphorylated SMAD1/5/8.

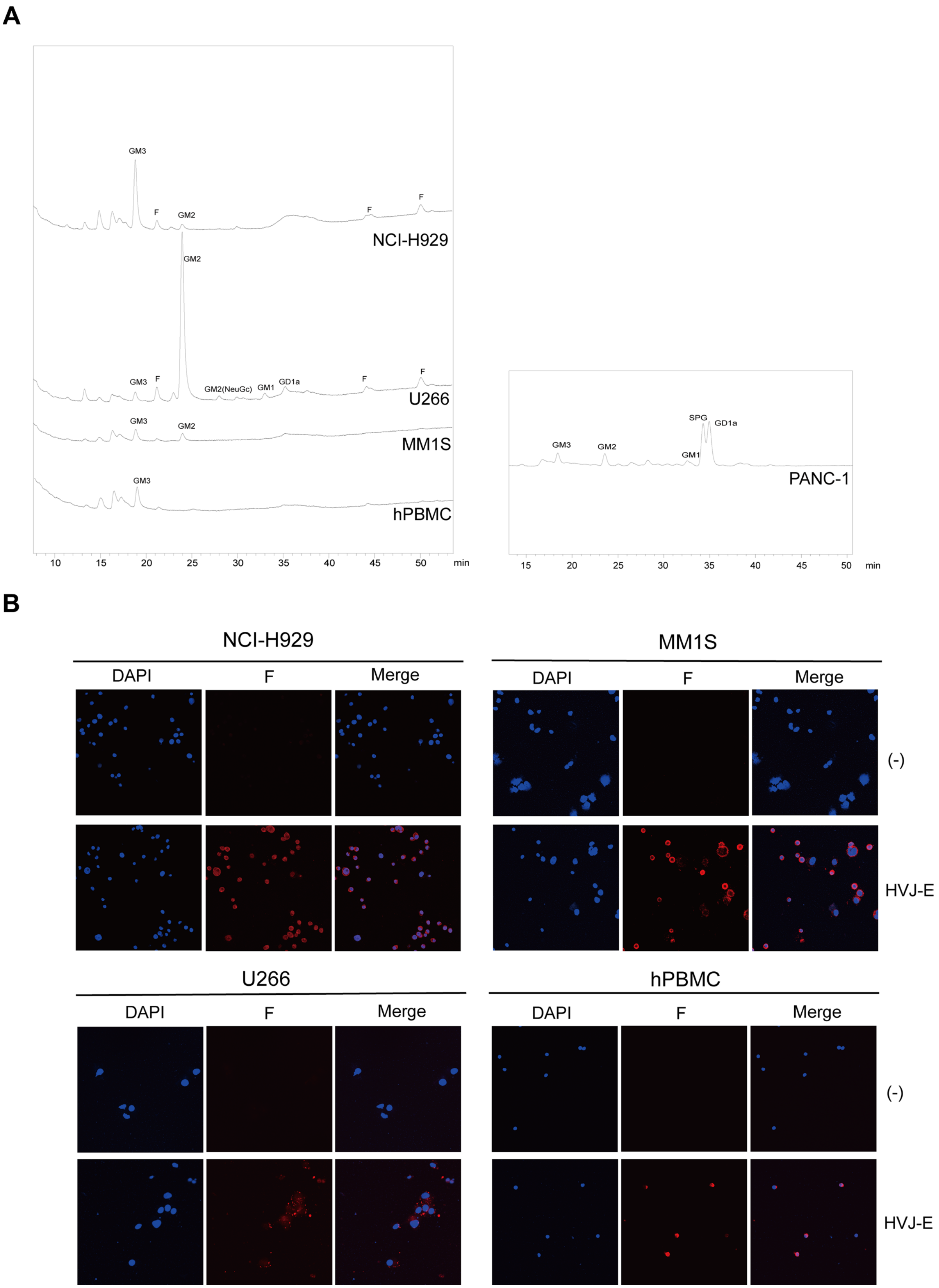

Supplementary Figure S8: HN glycoprotein-mediated binding to cells does not determine the interaction of HVJ-E with MM cells and hPBMCs from a healthy donor. (**A**) The expression pattern of gangliosides in MM cell lines (NCI-H929, U266 and MM1S), hPBMCs from a healthy donor and a human pancreas adenocarcinoma cell line (PANC-1) by HPLC analysis. (**B**) MM cell lines (NCI-H929, U266 and MM1S) and hPBMCs from a healthy donor were stained with anti-F protein (red) to visualize the binding between HVJ-E and the cells. The nuclei were stained with DAPI (blue).

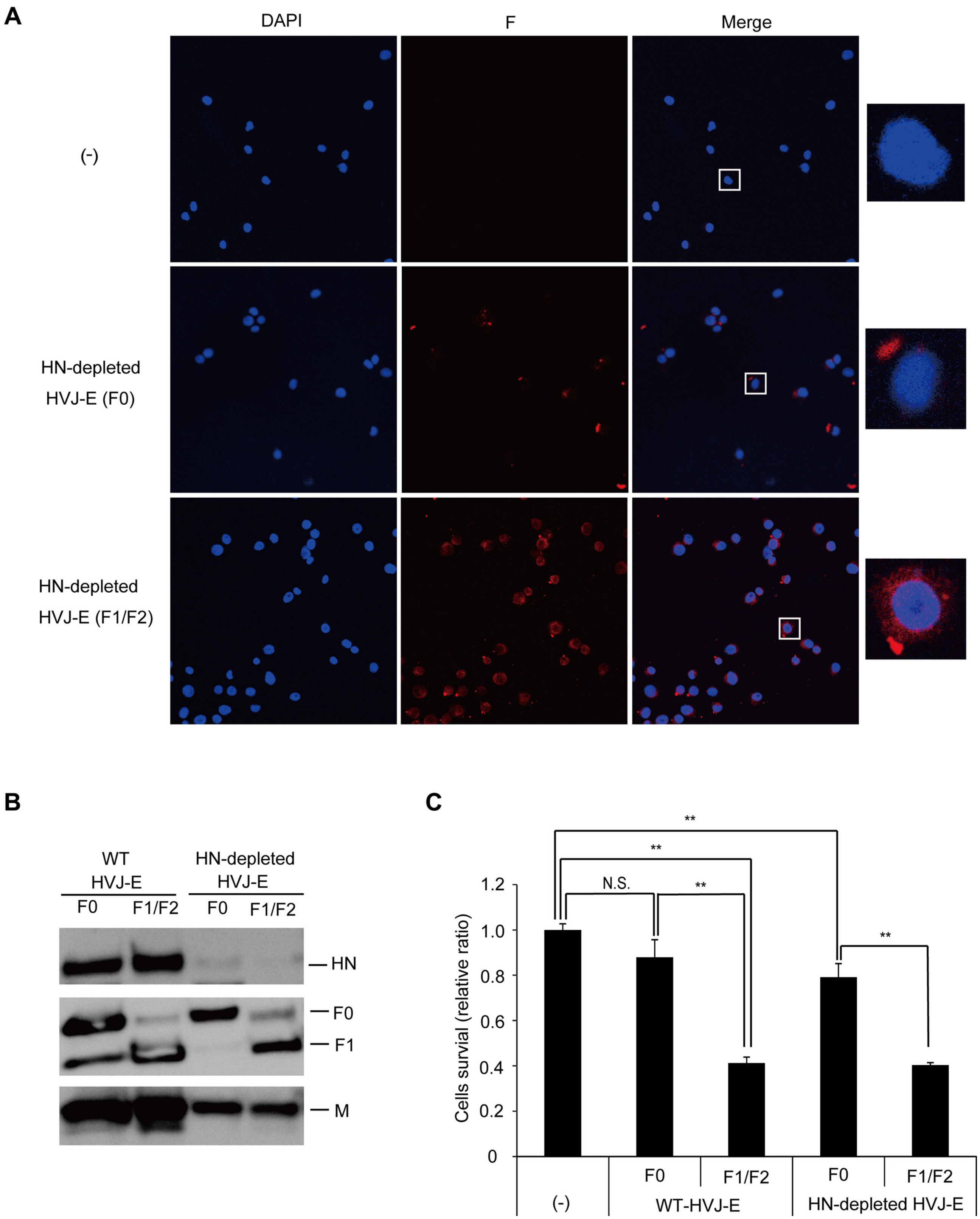

Supplementary Figure S9: Fusion glycoprotein activation determines the cytotoxicity of HVJ-E to MM cells. (**A**) The interaction of HN-depleted HVJ-E (F0 or F1/F2 form) with NCI-H929 cells was detected by immunostaining. NCI-H929 cells were stained with anti-F protein (red) to visualize the association between HVJ-E and cells, and the nuclei were stained with DAPI (blue). (**B**) Confirmation of viral protein (HN, F0, F1/F2 and M) expression in various types of HVJ-E by western blotting analysis. C. NCI-H929 cells were treated with the F-activated form (F1/F2) or -inactivated form (F0) of HVJ-E (wild-type or HN-depleted type) for 48 hours, and the cell viabilities were measured using the MTS assay. The data represent the mean ± SD of three independent experiments (***P* < 0.01, Tukey–Kramer test).

MATERIALS AND METHODS

Cell culture

Human umbilical vein endothelial cells (HUVECs) and human aortic endothelial cells (HAECs) were purchased from Lonza (Walkersville, MD, USA) and cultured in endothelial basal medium (EBM; Lonza) supplemented with EGM-SingleQuots (Lonza) and 10% FBS. All cells were used for experiments before passage 8. Monkey kidney cells (LCCMK2) were maintained in minimal essential medium (MEM; Nacalai Tesque) supplemented with 10% FBS, 100 U/ml of penicillin, and 100 μg/ml of streptomycin. The cells were incubated at 37°C in an atmosphere containing 5% carbon dioxide.

Antibodies

Bax (#5023), Bid (#2002), Puma (#4976) and Bim (#2919) were purchased from Cell Signaling Technology (Tokyo, Japan). CD3 (#300419), CD15 (#323029), CD14 (#325619) and CD19 (#302233) were purchased from BioLegend (San Diego, CA). Rabbit monoclonal antibodies (IgG) against the trans-membrane domain of the F protein, HN protein and M protein of HVJ-E were generated by Hokkaido System Science Co., Ltd. (Sapporo, Japan) using the peptide antigens of F, HN and M.

Transient transfection

In the Bim knockdown experiment, NCI-H929 cells were transfected with human Mission Bim (#1: SASI_Hs02_00341791; #2: SASI_Hs02_00341792; #3: SASI_Hs02_00341793), and Mission scramble siRNA served as the negative control (Sigma-Aldrich). HVJ-E RNA fragments were transfected into NCI-H929 cells by electroporation using the Neon® transfection system (Life Technologies) according to the manufacturer's instructions.

Generation of WT HVJ-E and HN-depleted HVJ-E

To generate wild-type (WT) HVJ-E, the culture medium from HVJ-infected LLCMK2 cells was passed through a filter and then centrifuged to precipitate the WT-HVJ particles. HN-depleted HVJ-E was produced as previously described. Briefly, HN-899 short interfering RNA (siRNA) was transfected into the LCCMK2 culture cells using Lipofectamine reagent. After another 48 hours, the culture medium was collected and filtered. HN-depleted HVJ particles were precipitated by centrifugation. F1/F2 WT-HVJ or F1/F2 HN-depleted HVJ was prepared by treatment with 5 μg/ml trypsin for 30 minutes at 37°C.

Analysis of acidic glycosphingolipids from human multiple myeloma cells and PBMCs from healthy donors

The majority of the experimental procedures have been previously reported. Briefly, the acidic glycosphingolipids were extracted from each MM cell line and PBMCs from healthy donors and digested with recombinant endoglycoeramidase II from Rhodococcus sp. (Takara Bio, Inc., Shiga, Japan). 2-Aminopyridine was used to label the released oligosaccharides because this reagent can be detected and separated using a high-performance liquid chromatography (HPLC) system equipped with a fluorescence detector. The molecular size of each PA-oligosaccharide is given in glucose units (Gu) based on the elution times of PA-isomaltooligosaccharides. Reversed-phase HPLC was performed on a TSK gel ODS-80Ts column (Tosoh). The retention time of each PA-oligosaccharide is given in glucose units based on the elution times of PA-isomaltooligosaccharides. Thus, a given compound on these two columns provides a unique set of Gu (amide) and Gu (ODS) values, which correspond to coordinates of the 2-D map. PA-oligosaccharides were analyzed using LC/ESI MS/MS. The standard PA-oligosaccharides PA-GM1 and PA-GD1a were purchased from Takara Bio, and PA-LST-a and PA-SPG were obtained from our previous study.

Immunofluorescence staining

MM cells or hPBMCs were seeded at 2×10^5 per well in 12-well plates. The cells were incubated with 10,000 MOI of wildtype HVJ-E or HN-depleted HVJ-E or PBS in 0.5 ml of culture medium for 1 hour. Next, the cells were washed and resuspended with D-PBS (-), and the cell suspension was loaded onto glass slides coated with a chemical linker (tissue capture liquid) and fixed in 4% paraformaldehyde for 10 minutes, followed by treatment with permeabilization buffer. After blocking with 10% goat serum, the cells were stained with a rabbit monoclonal antibody (IgG) against the F protein of HVJ-E and F236 overnight. Next, the cells were incubated with an anti-rabbit Alexa Fluor 546 antibody (Invitrogen) at room temperature for 1 hour to visualize the binding of HVJ-E to MM cells. DAPI was used for nuclear counterstaining. To detect intercellular p-Smad1/5/8 expression, NCI-H929 cells were stimulated with HVJ-E at a multiplicity of infection (MOI) of 1,000, with or without BAPTA-AM pre-treatment for 1 hour. After 8 hours of treatment, the cells were fixed in 3% paraformaldehyde for 10 minutes, permeabilized in 100% cold methanol for 5 minutes on ice, washed 3 times with PBS, blocked in PBS/0.3% Triton X-100/1% BSA/5% NGS for 1 hour at room temperature and incubated overnight with an anti-p-Smad1/5/8 antibody diluted 1:100 in PBS/0.1 Triton X-100/4% bovine serum albumin (BSA)/1% NGS at 4°C overnight.
